# AAV capsid bioengineering in primary human retina models

**DOI:** 10.1038/s41598-023-49112-2

**Published:** 2023-12-11

**Authors:** Adrian Westhaus, Steven S. Eamegdool, Milan Fernando, Paula Fuller-Carter, Alicia A. Brunet, Annie L. Miller, Rabab Rashwan, Maddison Knight, Maciej Daniszewski, Grace E. Lidgerwood, Alice Pébay, Alex Hewitt, Giorgia Santilli, Adrian J. Thrasher, Livia S. Carvalho, Anai Gonzalez-Cordero, Robyn V. Jamieson, Leszek Lisowski

**Affiliations:** 1 Translational Vectorology Research Unit, Faculty of Medicine and Health, Children’s Medical Research Institute, The University of Sydney, Westmead, Australia; 2 Infection, Immunity and Inflammation Teaching and Research Department, Great Ormond Street Institute of Child Health, University College London, London, UK; 3 Present Address: Genethon, Evry, France; 4 Eye Genetics Research Unit, Faculty of Medicine and Health, Children’s Medical Research Institute and Sydney Children’s Hospitals Network, The University of Sydney, Westmead, Australia; 5 Stem Cell and Organoid Facility, Faculty of Medicine and Health, Children’s Medical Research Institute, The University of Sydney, Westmead, Australia; 6 Lions Eye Institute, Nedlands, Australia; 7 Centre for Ophthalmology and Visual Sciences, The University of Western Australia, Nedlands, Australia; 8 Department of Anatomy and Physiology, The University of Melbourne, Melbourne, VIC Australia; 9 Department of Surgery, Royal Melbourne Hospital, The University of Melbourne, Melbourne, VIC Australia; 10 Menzies Institute for Medical Research, University of Tasmania, Hobart, Australia; 11 Centre for Eye Research Australia, University of Melbourne, Melbourne, Australia; 12 Stem Cell Medicine Group, Faculty of Medicine and Health, Children’s Medical Research Institute, The University of Sydney, Westmead, Australia; 13 Australian Genome Therapeutics Centre, Children’s Medical Research Institute and Sydney Children’s Hospitals Network, Westmead, NSW 2145 Australia; 14 Laboratory of Molecular Oncology and Innovative Therapies, Military Institute of Medicine - National Research Institute, Warsaw, Poland

**Keywords:** Molecular medicine, Preclinical research, Retinal diseases

## Abstract

Adeno-associated viral (AAV) vector-mediated retinal gene therapy is an active field of both pre-clinical as well as clinical research. As with other gene therapy clinical targets, novel bioengineered AAV variants developed by directed evolution or rational design to possess unique desirable properties, are entering retinal gene therapy translational programs. However, it is becoming increasingly evident that predictive preclinical models are required to develop and functionally validate these novel AAVs prior to clinical studies. To investigate if, and to what extent, primary retinal explant culture could be used for AAV capsid development, this study performed a large high-throughput screen of 51 existing AAV capsids in primary human retina explants and other models of the human retina. Furthermore, we applied transgene expression-based directed evolution to develop novel capsids for more efficient transduction of primary human retina cells and compared the top variants to the strongest existing benchmarks identified in the screening described above. A direct side-by-side comparison of the newly developed capsids in four different in vitro and ex vivo model systems of the human retina allowed us to identify novel AAV variants capable of high transgene expression in primary human retina cells.

## Introduction

Vectors based on adeno-associated viruses (AAVs) are the gene delivery vehicles of choice for in vivo gene therapies targeting many organs, including the human retina. The benefits of this vector system include efficient manufacturing, low pathogenicity, and, in most cases mild immunogenicity^1^. Applications of viral vector-mediated gene therapies hold great promise in immune-privileged compartments such as the central nervous system and the retina. The use of an AAV2 capsid to deliver a therapeutic cassette expressing a functional copy of the *RPE65* gene became the first approved retinal gene therapy to treat Leber congenital amaurosis (Luxturna, 2017, AAV2/2.ssAAV transgene)^2^. Expression cassettes packaged in the AAV2 capsid appeared to be efficiently delivered to the retinal pigment epithelium (RPE) at the site of injection when injected subretinally. However, AAV2 and other capsids were less successful at transducing retinal neurons across the neuroretina via subretinal or the surgically simpler intravitreal (IVT) injection routes, which prevents the applications of AAV2-based retinal gene therapies for diseases affecting photoreceptors and other retinal cells. Hence, there is a need to find novel AAV candidates that more successfully target human cells across the neuroretina^3^.

Significant efforts were undertaken by multiple research teams to develop novel capsids for efficient transduction of primary retinal cells following IVT administration. Notable examples of such novel bioengineered AAV variants include AAV-7m8, AAV-GL, and AAV-NN selected using murine photoreceptors and validated in canines and non-human primates^4,5^. More recently novel capsids were developed by directed evolution in non-human primate and canine retinal cells with the goal to move into clinical development^6,7^. To assess vector potency in human retinal tissue, natural and engineered AAV serotypes have been evaluated using human retinal explant cultures, well aligned with broadly accepted view on the importance of verifying AAV function in human primary tissue as a critical step towards clinical development^8^. However, despite this progress, to this day no novel AAV variants have been developed using primary human tissues, even though some candidates developed in murine, canine, and non-human primate photoreceptors have shown functional transduction of human tissue^4–10^. This study was set-up to investigate the suitability of primary human retina explants for novel AAV capsid development. Furthermore, while multiple AAVs for eye targeting have been reported, no unbiased comparison of the transduction performance across a large number of existing AAV vectors in various human retina models has been undertaken in the same study . Such a comparison, including both known retino-tropic variants as well as other variants not previously evaluated for retina transduction, could be very powerful as a strong retino-tropic variant may already exist among AAVs developed in unrelated applications.

To close this critical gap, this study was devised to include a comprehensive comparison of the transduction efficiency of over 50 existing AAV capsids in human primary retinal explants, human induced pluripotent stem cell (hiPSC)-derived retinal organoids^11,12^, primary human RPE, and hiPSC-derived RPE cells (Supplementary Fig. 1)^12^. The data generated from this screen were subsequently used to guide novel AAV variant bioengineering. To do so, directed evolution was performed using the functional transduction (FT) platform, which allows for a transgene expression-driven selection process^13^. The selection of novel AAV variants was performed in primary human retina explants and the selected variants were validated in the aforementioned human retina models as well as the murine retina. Consistent with the fact that the selection was performed using human primary retina, the studies showed that several of the novel selected variants were superior to the parental AAV2 capsid in the human models of the retina, but not in the murine model. Interestingly, the top variant from our screen, AAV2-M4, though it did not outperform AAV-7m8 in human retinal explants, showed greater tropism for human Müller glia cells. This work contributes towards a better understanding of whether ex vivo models such as the adult post-mortem human retina can serve as a platform for AAV directed evolution.

## Results

### Evaluating the performance of existing AAV variants in available models of the human retina

A published ‘AAV Testing Kit’^14^ containing an equimolar mix of 30 barcoded AAV variants was used to transduce human embryonic stem cell (hESC)—and hiPSC-derived retinal organoids. The AAV performance was analysed by next-generation sequencing (NGS) at the DNA (cell entry) and mRNA/cDNA (transgene expression) levels (Supplementary Fig. 2a). The DNA data showed very similar performance of most AAVs in the organoids generated from hESCs or hiPSCs with the exceptions of higher DNA contribution of AAV4 in the hESC-derived organoid and higher entry of AAV3b/LK03 in the hiPSC-derived organoid (Supplementary Fig. 2b). Overall, the most efficient AAV capsid variants at cell entry in the organoid were AAV13, AAV-DJ and AAV-7m8 (Supplementary Fig. 2b, hESC and hiPSC DNA).

All NGS data was adjusted with respect to the input NGS data of the vectorized AAV capsid mix. On the level of transcriptional activity, similar patterns regarding the AAV4 and AAV3b/LK03 discrepancy between the models were observed. Overall, the highest RNA contribution was attributed to AAV-7m8, followed by AAV-DJ and AAV-NP66.

Based on the results obtained from the ‘30 AAV Kit’ study, a larger and more comprehensive study was designed. An AAV Kit containing an additional 21 AAV variants was generated, with the overall goal to increase the phylogenetic diversity of the AAVs that were compared. The 21 novel variants added were based on several different parental variants, were generated using different techniques, and were selected in different cells and tissues (Supplementary Table 1). Additionally, to increase robustness of the AAV screen, every AAV variant in the new ’51 AAV Kit’ was included with two unique barcodes, so that the final Kit contained 102 individual AAV preps (51 AAV variants × 2 barcodes per variant) that were titered and mixed at 1:1 molar ratio.

**Figure 1:**
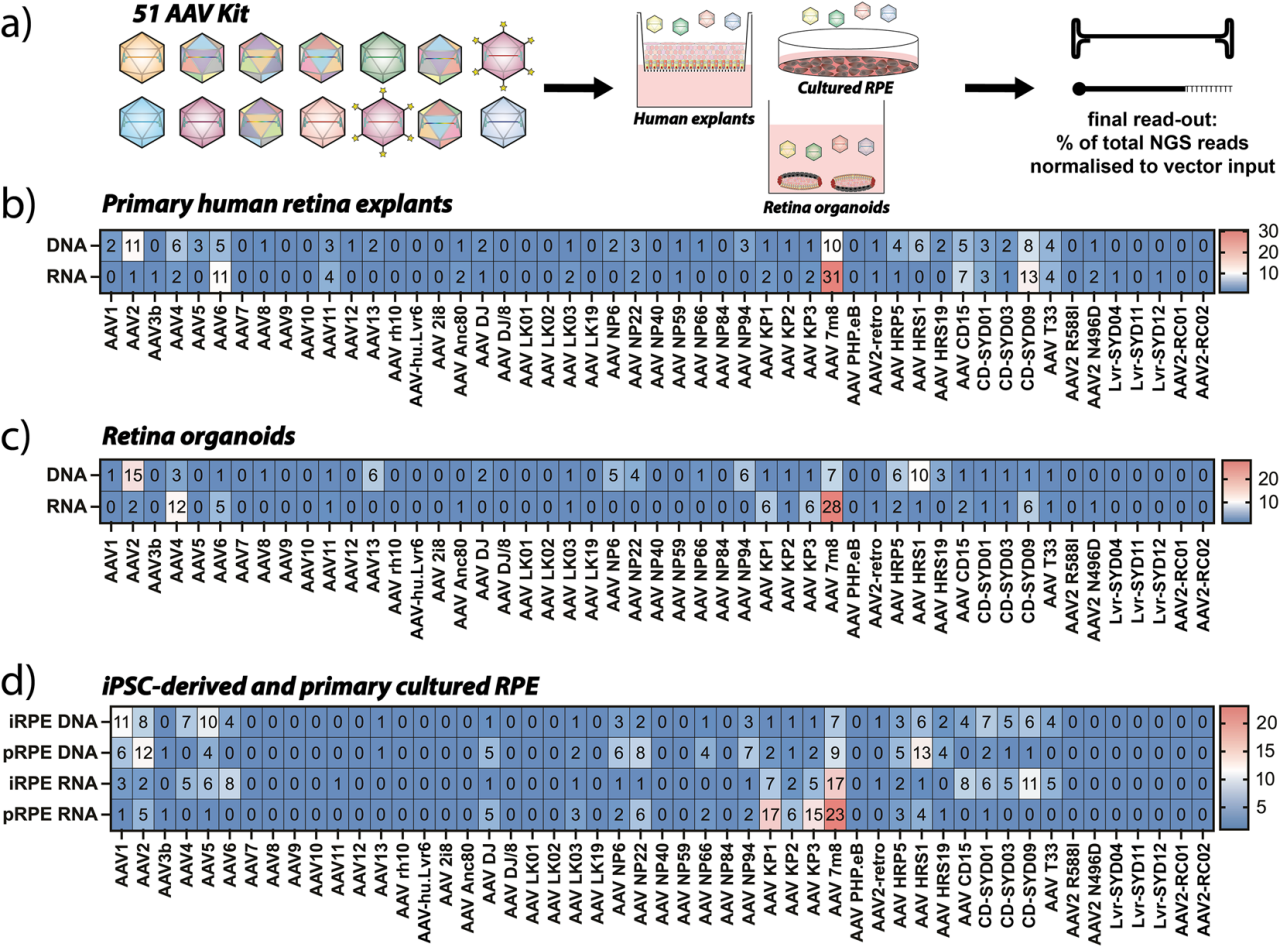
The 51 ‘AAV Kit’ in a range of models of the human retina. (**a**) Schematic representation of the performed experiments using the double-barcoded eGFP AAV Kit. (**b**) Results of NGS-based quantification of vector-encoded DNA and RNA/cDNA in retina explants. (**c**) Results of NGS-based quantification of vector-encoded DNA and RNA/cDNA in retinal organoids. (**d**) Results of NGS-based quantification of vector-encoded DNA and RNA/cDNA in retinal pigment epithelium (RPE). Values are given as percentage of total reads by making the sum of both barcodes and averaging across independent experiments. Number of independent experiments: Explants: n = 1; Organoids: n = 2; iRPE: n = 4; pRPE: n = 1.

This novel ‘51 AAV Kit’ was used to transduce polarized primary human explants in interphase culture (Fig. 1b), hiPSC-derived retinal organoids (Fig. 1c) and cultured RPE cells either derived from hiPSCs (iRPE, Fig. 1d) or primary tissue (pRPE, Fig. 1d). The human retina explants were cultured in transwells with the photoreceptor layer facing down and the AAVs added from the vitreal side (inner limiting membrane/retinal ganglion cell layer) to recapitulate intravitreal injection conditions^5,15^. The values in Fig. 1b–d are shown as sums of the contributions from two barcodes per AAV variant, as this method was shown to yield highly robust data in previous studies^13^.

The highest DNA contribution was achieved by AAV2, followed by the retina capsid AAV-7m8 and the shuffled capsid AAV-CD-SYD09 (high AAV6 contribution) that was selected in human hematopoietic stem cells^16^. On the transcriptional level, AAV-7m8 was the strongest transducer followed again by AAV-CD-SYD09 and the closely related AAV6 (Fig. 1b).

In hiPSC-derived retinal organoids, we observed the strongest DNA contribution from AAV2 followed by AAV-HRS1 (selected from a DNA shuffled library on HuH-7 cells^16^) and AAV-7m8, both of which have high homology with AAV2. On the RNA level, AAV-7m8 was by far the strongest variant, followed by AAV4 and AAV-KP1^17^ and AAV-KP3^17^ (Fig. 1c).

Lastly, two different models of the human RPE (iRPE cells derived from hiPSCs and primary tissue pRPE) were transduced with the ‘51 AAV Kit’ and analyzed by NGS. The variants entering iRPE most efficiently were AAV1, AAV5, and AAV2, while pRPE had the highest DNA contribution from AAV-HRS1, AAV2, and AAV-7m8 (Fig. 1d). On the level of RNA expression, iRPE was most efficiently transduced by AAV-7m8, AAV-CD-SYD09, and AAV6/AAV-CD15^16^, while the pRPE was most efficiently transduced by AAV-7m8, AAV-KP1 and AAV-KP3.

### Using the FT platform to select novel retino-tropic AAVs

**Figure 2:**
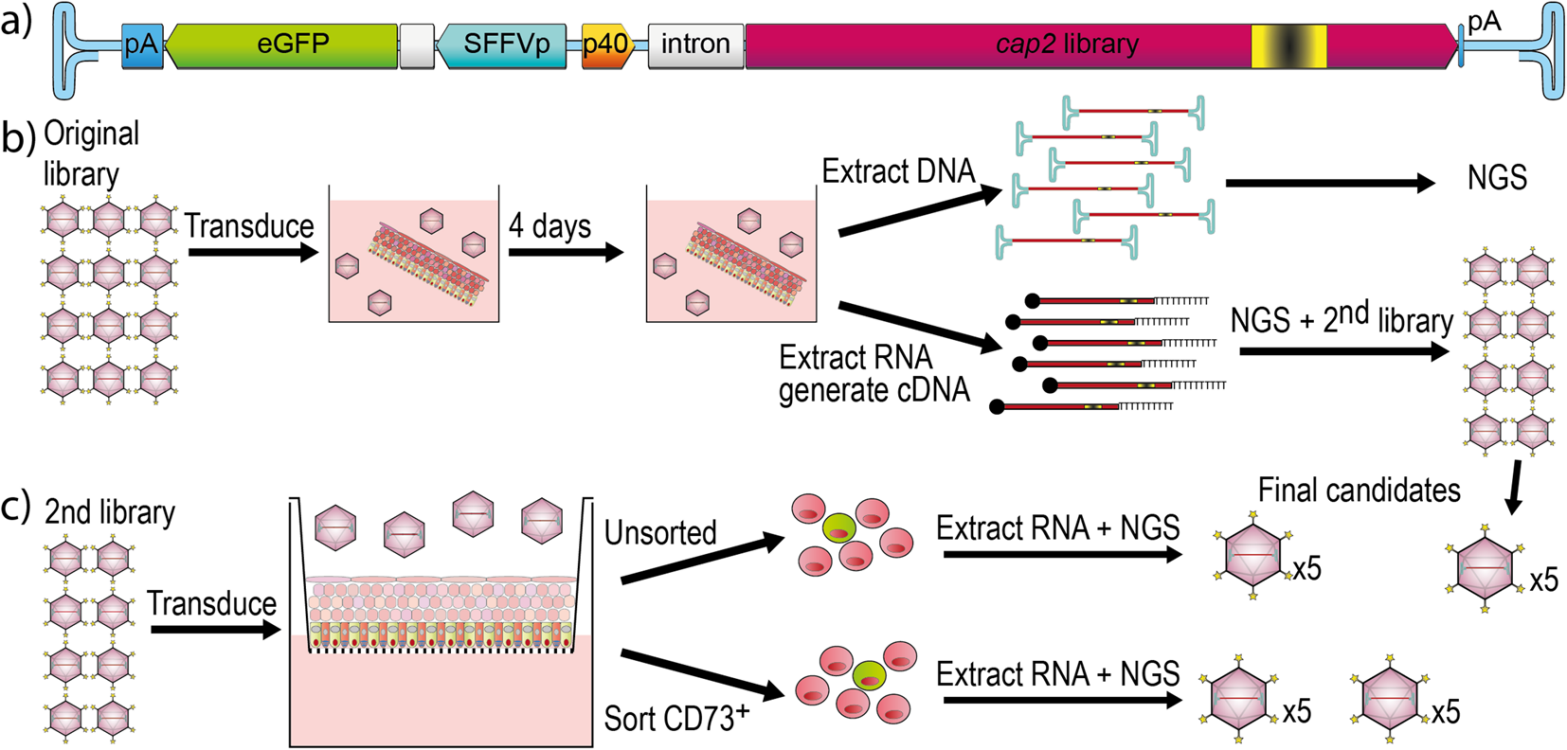
Schematic of the capsid screening approach in primary human retina explants. (**a**) Graphical representation of the Functional Transduction platform^13^ used to select novel retina-tropic capsids based on high RNA expression. (**b**) The original library was used to transduce hole-punches of explanted primary human retina. DNA and RNA were extracted, and the RNA was used to create the secondary library. (**c**) The secondary library was used to transduce the inner nuclear layer of polarized human retina explants. Tissue was processed for whole RNA extraction or extraction from CD73^+^ outer nuclear cells to enrich for AAV capsids that can cross the retina. Sequences from recovered RNA from both rounds was used to generate 20 novel candidates for validation.

We previously established and validated a FT platform, which enables directed evolution to select novel AAV capsids based on transgene expression^13^. Next, we used this platform to perform selection of an AAV2-based peptide display library on primary human retina explants (Fig. 2a). AAV2 was used as the parental serotype in this study as it is approved for clinical use in the retina and because AAV-7m8 (an AAV2-based peptide insertion variant) was the most successful candidate in the first part of the study.

The selection was performed as a two-round process, starting with an initial enrichment for functional capsids by transducing ‘free-floating’ retina explants. Based on our experience, these ‘free-floating’ explant cultures have a higher viability and make it easier for the vector to access and thus transduce the cells. We hypothesized that using a less stringent system was critical given the large variability of the AAV library (4,200,000 variants based on NGS analysis) and thus the low effective dose per cell of individual variants and would lower the risk of losing good candidates that were in low abundance in the original library. Four days after exposure to the library (limiting the total culture period to 5-day to ensure tissue integrity), the explant was harvested, and DNA and RNA were extracted. Amplicons from both total DNA and RNA/cDNA underwent NGS analysis. The results showed an over 80-fold stronger enrichment for RNA/cDNA recovery compared to DNA recovery (~ 750,000 peptide variants remained from an initial 4,200,000 peptide variants left for the DNA-based recovery, while only ~ 9000 peptide remained for RNA/cDNA recovery). Due to the much greater stringency, only the RNA/cDNA selection was moved to the second round (Fig. 2b).

The transgene expression-enriched library from round one was used to transduce polarized human retina explants in transwell cultures (Fig. 2c)^15^. After dissection, the retina was separated into quarters and one quarter was cultured per well. The photoreceptor layer was placed on the transwell membrane and AAVs were applied to the vitreal (ganglion cell layer) side. The library was applied at four doses (see Methods, Library selection—Round 2), and the transduction was allowed to proceed for four days prior to harvest and processing. To increase the chance of selecting capsids that were able to migrate through all the layers of the retina and transduce photoreceptors, three quarters of the tissue (the two pooled medium doses and the highest dose conditions) were sorted for CD73-expression, a rod photoreceptor cell marker. The remaining unsorted tissue (lowest library dose) was also analyzed as it could lead to the identification of AAV candidates transducing other clinically relevant cells of the retina. Following the NGS analysis of the cDNA amplicons, five top performing AAV candidates were selected from samples transduced with high (AAV2-H1 to H5), medium (AAV2-M1 to M5), and low (AAV2-L1 to L5) doses of the library. We also included five top candidates from the first round of selection (AAV2-1.1 to 1.5) creating a panel of 20 novel capsids for further validation (Fig. 2c, see Supplementary Table 3 for full list of peptide sequences).

### Validation of novel capsids in various models of the retina

**Figure 3:**
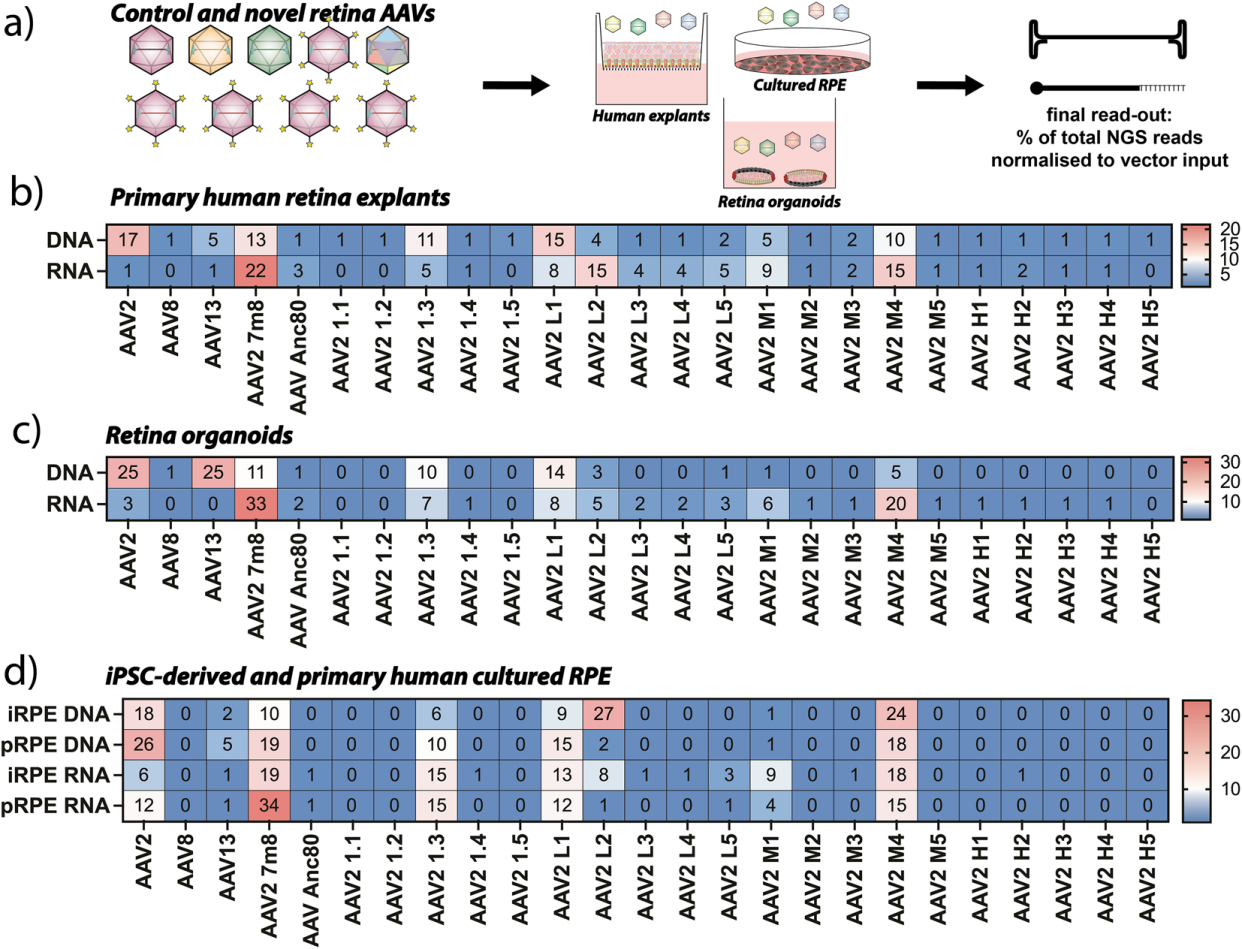
Performance of the novel retina-tropic capsids in a range of models of the human retina. (**a**) Schematic representation of the performed experiments using 20 novel retina capsids and five existing benchmarks as a double-barcoded eGFP Retina AAV Kit. (**b**) Results of NGS-based quantification of vector-encoded DNA and RNA/cDNA in retina explants. (**c**) Results of NGS-based quantification of vector-encoded DNA and RNA/cDNA in retinal organoids. (**d**) Results of NGS-based quantification of vector-encoded DNA and RNA/cDNA in retinal pigment epithelium (RPE). Values are given as percentage contributions of total reads by making the sum of both barcodes and averaging across independent experiments. Number of independent experiments: Explants: n = 2; Organoids: n = 2; iRPE: n = 6; pRPE: n = 2.

The 20 novel capsids were vectorized and used to package our previously validated barcoded cassette encoding enhanced green fluorescent protein (eGFP) under the control of the cytomegalovirus (CMV) promoter^14^. Similarly to the ‘51 AAV Kit’, to increase data stringency each AAV variant was used to package two barcoded cassettes, creating an internal control for each AAV. Based on our results (Fig. 1) as well as published work^18^, AAV2, AAV8, AAV13, AAV-7m8, and AAV-Anc80 were included as controls. In addition to retina explants (the model on which the novel AAVs were selected), hiPSC-derived retinal organoids, iRPE, and pRPE were also included in our novel capsid validation studies (Fig. 3a).

Transduction of the human retina explants in the polarized transwell cultures showed that the DNA contribution was highest from AAV2 followed by the novel candidate AAV2-L1 and the control AAV-7m8 (Fig. 3b). However, AAV2 was not able to lead to effective transgene expression. The strongest transgene expression was observed for AAV-7m8, followed by AAV2-M4 and AAV2-L2 (Fig. 3b).

To perform functional testing of the novel variants in an in vitro model that can be cultured for an extended period of time, we transduced retinal organoids with the same mix of novel retina AAVs. A similar pattern was observed in hiPSC-derived retinal organoids, where AAV2 and AAV13 were the most effective at the DNA level followed by AAV2-L1. However, at the RNA level, AAV-7m8 outperformed all the variants, with AAV2-M4 and AAV2-L1 taking the 2nd and 3rd spots (Fig. 3c). For RPE cells, the best performing capsids at the DNA level were AAV2-L2, AAV2-M4, and AAV2 for iRPE and AAV2, AAV-7m8, and AAV2-M4 for pRPE. On the RNA level, AAV-7m8 performed best followed by AAV2-M4 and AAV2-1.3 for both iRPE and pRPE (Fig. 3d).

Overall, the NGS results from all models suggest that while novel candidates such as AAV2-1.3, AAV2-L1, AAV2-L2, AAV2-M1, and AAV2-M4 outperform their parental AAV2, the AAV-7m8 is the best performing AAV candidate among the panel tested.

### Side-by-side comparison of AAV-7m8 and AAV2-M4 in human retina models

**Figure 4:**
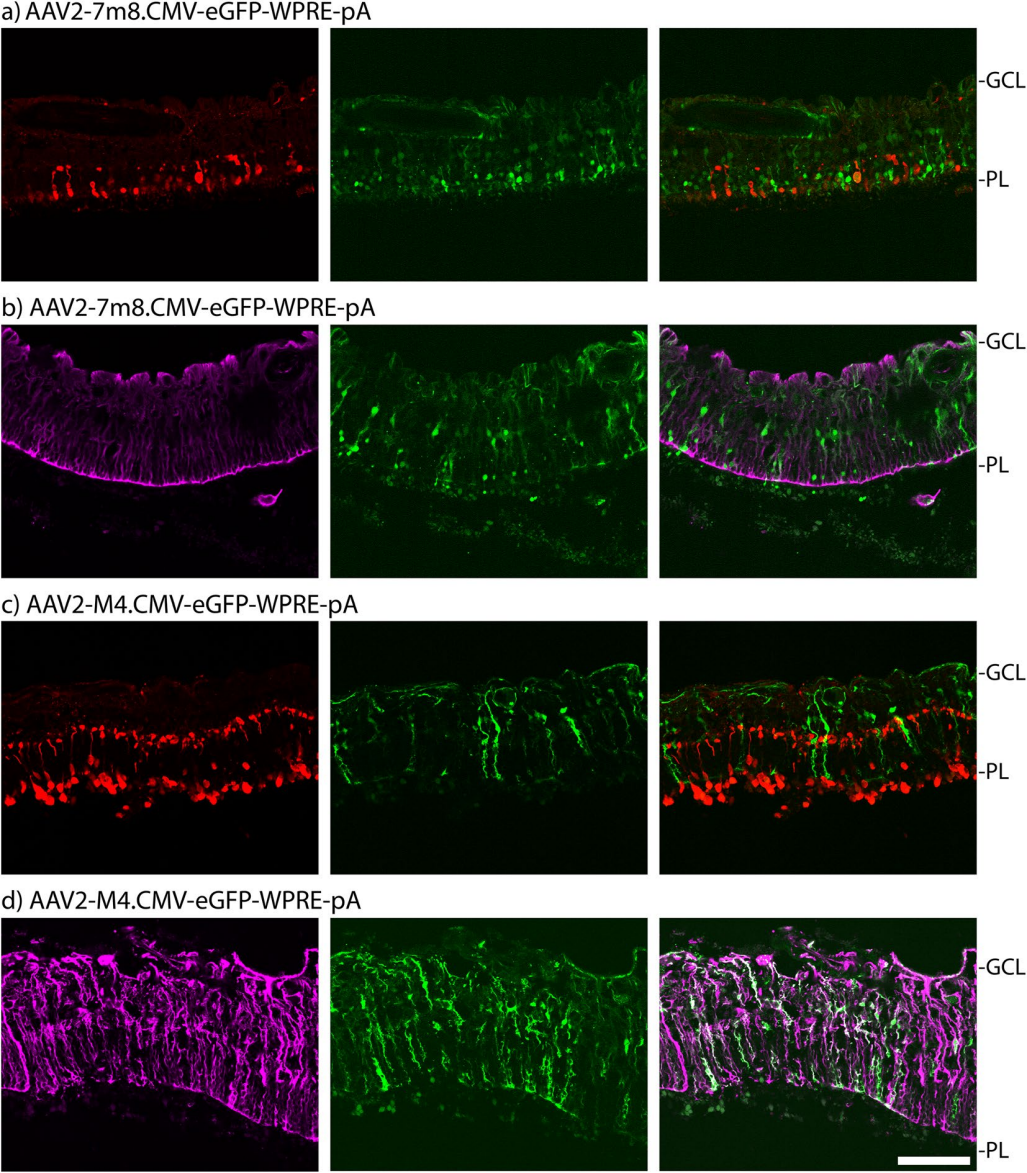
Side-by-side analysis of AAV-7m8 and AAV2-M4 in retina explants. Transduction of polarized retina explants with 5 × 10^10^ vector genomes using AAV-7m8 and AAV2-M4; n = 1 (**a**) AAV-7m8 transduction showing confocal images with antibody-labelled cone arrestin (red), native CMV-driven eGFP expression (green), and an overlay of both, from left to right. (**b**) AAV-7m8 transduction showing confocal images with antibody-labelled vimentin (purple), native CMV-driven eGFP expression (green), and an overlay of both, from left to right. (**c**) AAV2-M4 transduction showing confocal images with antibody-labelled cone arrestin (red), native CMV-driven eGFP expression (green), and an overlay of both, from left to right. (**d**) AAV2-M4 transduction showing confocal images with antibody-labelled vimentin (purple), native CMV-driven eGFP expression (green), and an overlay of both, from left to right. The scale bar in (**d**) is 100 µm and applies to all images. *Abbreviations* GCL, Ganglion cell layer; PL, Photoreceptor layer.

The NGS-based vector comparison provides an insight into a relative vector performance based on average signal from all the cells in the processed sample. To gain a better understanding of the novel AAV vectors performance in the individual cell types within the complex preclinical retina model systems, we set out to study the specific cell type tropism of AAV2-M4. AAV-7m8 was used as a control. To do so, we first individually transduced the polarized human retina explants with the two capsids packaging the same CMV-driven eGFP cassette used in experiments reported in Figs. 1a and 3a. Four days after transduction, the tissues were fixed and used for immunofluorescence analysis. Analysis of tissue sections stained with a cone specific anti-arrestin antibody showed that AAV-7m8 transduced cones as well as many other cell types in the human retina explants (Fig. 4a). In contrast, AAV2-M4 only transduced elongated, filamentous cells within the center of the retina, rather than cells in the photoreceptor layer (Fig. 4c).

As the cells targeted by AAV2-M4 appeared to morphologically resemble Müller glia cells, samples were further analyzed using an anti-vimentin antibody. While AAV-7m8 showed only a minimal level of co-staining with vimentin (Fig. 4b), AAV2-M4-mediated eGFP expression showed a high co-staining with Müller glia cells (Fig. 4d).

Next, we evaluated if the same transduction pattern could also be observed in retinal organoids. Interestingly, in this preclinical model transduction of AAV-7m8 was restricted to Müller glia cells (Supplementary Fig. 3a) and no labelling of cone cells was observed (Supplementary Fig. 3b). AAV2-M4 labelled Müller glia cells with high efficiency (Supplementary Fig. 3c) and specificity as shown by co-labelling of the Müller glia marker cellular retinaldehyde-binding protein (CRALBP, Supplementary Fig. 3d).

Given that none of the novel AAVs as well as AAV-7m8, an AAV known for high efficiency in photoreceptor cells, were able to label cones in the organoid model was unexpected and we hypothesized that the CMV promoter might not be optimal for reporter expression in photoreceptors in these organoid cultures. We performed a study using the previously published glia de-targeted human synapsin1-AAV-p40 chimeric promoter (in a *scAAV-human synapsin 1 reverse (hSYNrv)-AAVp40-eGFP-pA* transgene) packaged in AAV-7m8^13^. The data showed that AAV-7m8 was able to functionally transduce photoreceptor cells and interneuron cells when using this promoter, while the Müller glia cells remained untransduced as shown as a lack of co-staining with the Müller glia marker CRALBP (Supplementary Fig. 4a). We observed a similar profile for AAV2-M4 with the same promoter, including the absence of co-labelled CRALBP and eGFP positive cells, although there was a stronger bias towards labelling interneurons rather than photoreceptors (Supplementary Fig. 4b). Based on these results we subsequently used this promoter construct to functionally compare AAV-7m8 and AAV-M4 by transducing three independent organoid cultures. Using flow cytometry, we quantified the transduction efficiency in cell types that showed labelling with the *hSYNrv-AAVp40* promoter. The percentage of DAPI-negative eGFP positive cells (mix of photoreceptors and interneurons based on the images in Supplementary Fig. 4a,b) revealed that both capsids were equally efficient at transduction (n = 3 batches of differentiations five organoids each, Supplementary Fig. 4c).

### Validation of the top candidates in iRPE

The RPE cultures used in the NGS studies differ from the other models, as these are not consisting of a complex mix of cell types but should only include a single cell population. Specifically, while the primary RPE are mostly homogenous, the hiPSC-derived RPE may have different populations of varying maturity. To test this hypothesis and confirm that we observe true RPE transduction in the iRPE cultures, we exposed hiPSC-derived RPE to individual AAV variants encoding the single stranded CMV-eGFP cassette and compared the transduction efficiencies by flow cytometry, which also enabled the morphological differentiation of more granular differentiated and smoother (hiPSC-like) immature cells. The AAV variants selected for this experiment were those with high performance in both iRPE and pRPE, except for AAV2-L2 which performed well in iRPE but not pRPE. AAV-shH10 was included in this study as it was previously shown to transduce human stem-cell-derived RPE with very high efficiency^12^. Interestingly, the harvested culture showed a difference in morphology of the DAPI-negative cells (Supplementary Fig. 5a). Defining the cell population with the higher side scatter (SSC) as the granular/complex more mature cells and the population with the lower SSC as the less mature smooth population, three separate datasets of transduction efficiencies were generated.

Interestingly, the transduction efficiency was generally higher in the complex populations indicating that the more mature cells may be less resistant to AAV transduction than the immature (hiPSC-like) cells (Supplementary Fig. 5b,c). This difference was particularly apparent for the cells transduced with AAV2-M4, which had the lowest efficiency in immature cells but showed a high eGFP percentage and the highest eGFP mean fluorescence intensity in the granular, mature RPE cells.

### Evaluation of novel AAV variants in the murine retina in vivo

**Figure 5:**
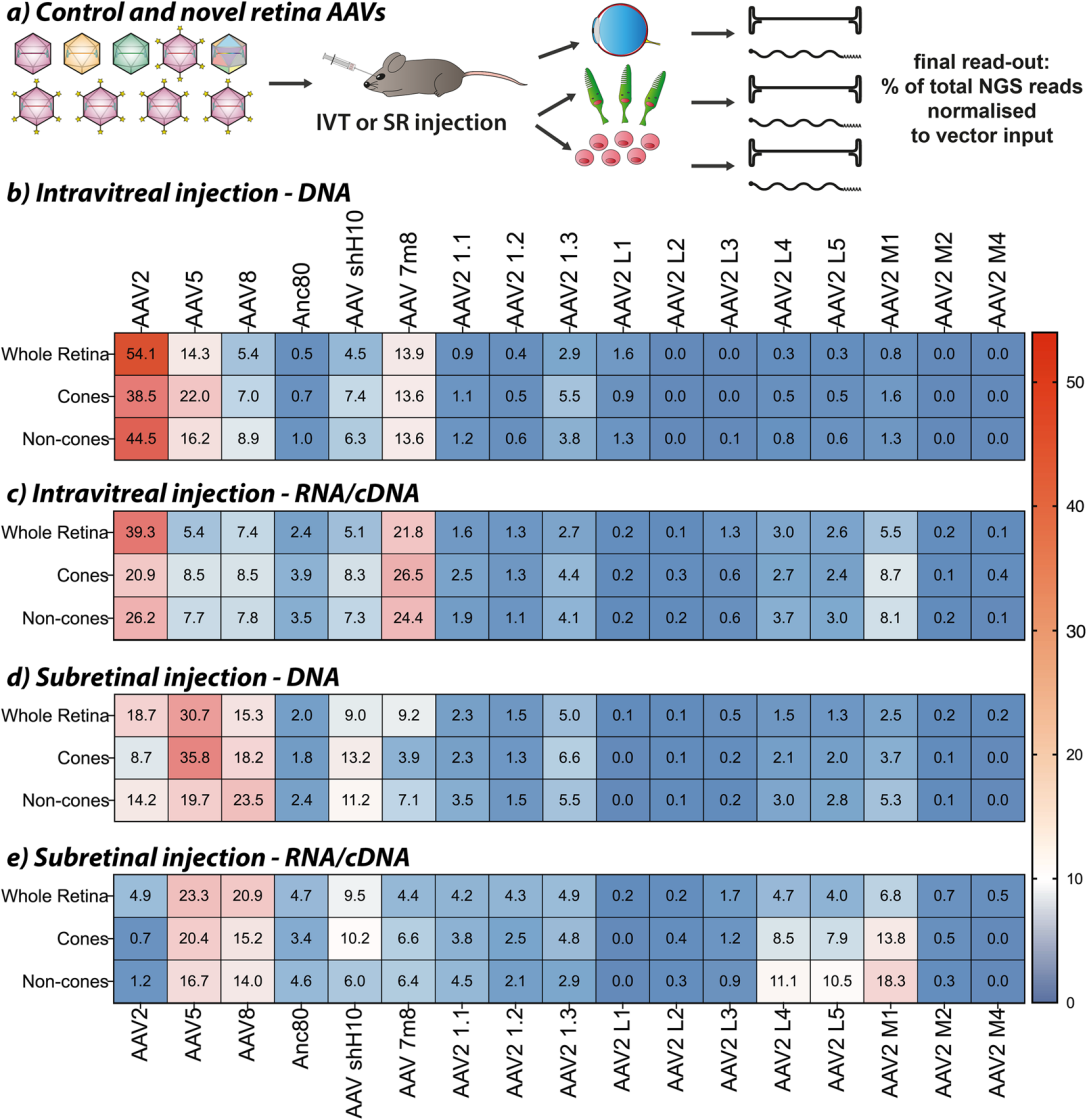
Evaluation of novel AAV variants in the murine retina. (**a**) The triple-barcoded mCherry ‘Retina AAV Kit version 2’ was injected into mouse eyes either via the intravitreal (IVT) or the sub-retinal (SR) route. Four weeks after injection, the retina was harvested, and cells were sorted for eGFP expression from murine cones. Whole retinas, sorted cones, and ‘non-cones’ were used for DNA and RNA extraction followed by NGS analysis. (**b**,**c**) Percent of read contributions in murine retina cells from extracted DNA [b] and RNA/cDNA [c] following IVT injection. (**d**,**e**) Percent of read contributions in murine retina cells from extracted DNA [d] and RNA/cDNA [e] following SR injection. All experiments were performed in three murine eyes from three separate animals each.

Although our main requirement for the novel AAV capsids was to efficiently transduce human retina cells, the translational power of the variants would be improved if they could also be utilized in preclinical models. To this end we assessed if they also showed high efficiency in the murine retina. Therefore, murine eyes were injected with a subset of novel AAVs (only variants that worked in previous studies and could be manufactured to scale). We also added AAV5 and AAV-ShH10^19^ to the panel of control AAVs. To facilitate analysis of AAV transduction specifically to cone photoreceptor cells, we used a transgenic mouse model where the cones constitutively express eGFP under the Chrnb4 (cholinergic receptor nicotinic beta 4 subunit) promoter. Thus, the selected novel AAVs and control variants were used to package a barcoded CMV-mCherry cassette. Each AAV was used to package three individual and uniquely barcoded cassettes, allowing us to simultaneously obtain three independent data points for each vector, thus increasing robustness of the data generated with this triple barcoded AAV Kit. The equimolar mix of 17 AAVs (11 new variants and 6 controls) was injected via IVT and SR routes and DNA and RNA/cDNA was extracted from whole retinas, sorted cones, and sorted ‘non-cone-cells’ 28 days after vector delivery (Fig. 5a).

NGS analysis showed that AAV2, AAV5 and AAV-7m8 were the most efficient at cell entry when delivered via the IVT route (Fig. 5b). The highest transgene expression in the whole retina as well as ‘non-cones’ was observed for AAV2, AAV-7m8 achieved the highest expression in sorted cones, while AAV5 played no major role at the level of transgene expression (Fig. 5c). AAV2-L1, AAV2-L2, and AAV2-M4, which previously showed strong transduction in human retina cells (Fig. 3b), were not very efficient at transducing mouse retina both at the DNA and RNA/cDNA levels. The only variants selected on human cells that also worked with moderate efficiency in the mouse model were AAV2-M1 and AAV2-1.3. Interestingly, AAV2-M1 was the third-best transducer at the RNA/cDNA level for sorted cones showing a remarkable increase from a very low DNA contribution (Fig. 5b,c).

The strongest entry into murine retina cells via the SR injection was observed for AAV5, AAV8, AAV2 and AAV-ShH10 while AAV-7m8, AAV2-1.3 and AAV2-M1 showed only moderate performance at the cell entry level (Fig. 5d). On the level of transgene expression, AAV5, AAV8, and surprisingly AAV2-M1, showed the strongest performance in murine retina cells. Interestingly, other novel variants such as AAV2-L4 and AAV2-L5 showed a moderate transduction of sorted ‘non-cone cells’, even though these variants were not previously found to perform well in the human models (Fig. 5e).

While there are many potential reasons why AAVs performing well in the human retina explant perform poorly in murine retina in vivo, one of the obvious differences between those two models is the presence of an immune system. To gain an insight into the immunological profile of the novel AAV variants, we performed neutralization assays using pooled intravenous immunoglobin (IVIg). After establishing a working dose for the novel variants, as well as control AAV2 and AAV-7m8, in HeLa cells (Supplementary Fig. 6a,b), we performed the neutralization assay. Studies using three IVIg concentrations showed that all novel variants had similar neutralization profiled to AAV2, while AAV-7m8 was more resistant to neutralization than AAV2 (Supplementary Fig. 6c).

## Discussion

In the presented study, a large number of AAV variants was compared in several models of the human retina. Additionally, the fit-for-purpose FT-platform was used to select novel bioengineered AAV capsids that outperformed the parental AAV2 capsid. The choice for AAV2 as the parental capsid was largely driven by the clinical use of AAV2 and the strong performance of AAV-7m8, an AAV2-based variant. Furthermore, the vast majority of recent capsid selections in the retina featured AAV2-derived variants as their top candidates, even when comparing different types of libraries^5,6^. The top novel candidates identified were particularly promising candidates for therapeutic cargo delivery to the RPE and Müller glia cells. In our study, cone photoreceptor cells, another clinically important target, were best transduced by AAV-7m8. Importantly, we showed that the novel candidates, such as AAV2-L2 and AAV2-M4, we selected on primary human retinal cells showed selective tropism towards the human cells and showed minimal transduction of murine cells in vivo.

It was anticipated that AAV-7m8 would perform well among tested variants in stem cell-derived organoids given how well AAV-7m8 performed at the RNA level in previous experiments using hiPSCs^14,20^ and the fact it was selected for the transduction of the mouse retina^4^. Interestingly, aside from the dominance of AAV-7m8, several differences in how the retinal organoids were transduced by the ‘51 AAV Kit’ were observed compared to the data previously obtained by us using a ‘30 AAV Kit’^14^.

One possibility for the observed discrepancies is that, despite the fact that both experiments used hiPSC-derived organoids, the organoids were generated from different hiPSC lines and in different facilities. As the experiments with the ’30 AAV Kit’ revealed that there are slight differences between hESC and hiPSC-derived organoids, the origin of the organoid can have a strong influence on the AAV performance, which could explain the observed differences in functional AAV transduction between both studies. Yet despite the differences, AAV-7m8 was the highest expressing variant in all organoids tested, indicating a robust transduction profile of this variant in those cells.

Importantly, the data obtained with the ‘51 AAV Kit’ as well as the novel retina variants for transduction of human retina explants showed a substantial overlap with the data obtained for human retinal organoids. While the organoid model was not able to recapitulate the polarity and orientation of the cultured human explant, the functionality of AAV variants appeared to be very similar between both models. Similarly, the overlapping data between iRPE and pRPE models suggests that the iRPE protocol yields pigmented epithelial cells that are similar enough to allow functional AAV testing for human RPE.

The NGS results obtained from the entire explant or organoid are a good indication of the overall average vector performance, however, are unable to resolve the differences in individual vector performance in specific cellular subsets. A recent study pioneered the use of single cell RNA sequencing technology to elucidate this^8^. The reported screen was performed in retinal explants and comprised 18 AAV variants and demonstrated the robust performance of AAV-K91 that was developed in the canine retina. It would be of great interest to see how well the results of that screen translate into stem cell-derived and in vivo models.

On the cellular level in our results, the immunofluorescence staining showed that AAV-7m8 could transduce all cells of the retina, while AAV2-M4 and other novel capsids (data not shown) were limited to the glia cells. Therefore, while AAV-7m8 could achieve a higher overall combined RNA expression from various cell types, AAV2-M4 was a more specific and stronger expresser in a very specific subset of cells. However, it is important to note that NGS-based comparisons are a very powerful method but only to gain a first insight into the relative performance of a larger panel of variants in a direct side-by-side comparison. Individual variant follow-up studies, such as those presented in Fig. 4, are subsequently needed to understand cellular tropism in a complex tissue. Additionally, it is important to note that the NGS data generated with all the models included in this study does not give us an insight into whether or not the AAV vectors will work after in vivo delivery in human patients as important barriers of the human eye are not present in these in vitro models.

The current data does not allow us to determine whether the lack of photoreceptor transduction by AAV2-M4 in the explant was due to the inability of the vector to cross through the retina to reach photoreceptors or if it was a bona-fide selective tropism for Müller glia cells. The latter possibility appears unlikely, as we saw low transduction of photoreceptors and high transduction of interneurons in retinal organoids using the glia de-targeted hSYNrv-AAVp40 promoter.

As we did not see this phenotype in other model tissues and only with the glia-detargeted AAV-p40 promoter^13^, it could be possible that AAV2-M4 transduced cells differently depending on the promoter as previously described for other AAV variants in preclinical brain models^21^. Of note, AAV2-M4 was a candidate that was recovered from CD73-positive cells, which we hypothesized would have ensured a strong photoreceptor tropism of selected AAVs. While it is challenging to explain this experimental outcome, one possible hypothesis is that the Müller glia cells were co-purified together with the CD73-positive photoreceptors during the cell sorting process. Despite the inconsistencies between the intended outcome of the selection process and the actual cellular tropism of AAV2-M4, it was included in further studies, due to its interesting tropism.

AAV-7m8, and in following studies AAV2-M4 and AAV2-L2, were consistently strong transducers in human models of various retinal cells, indicating that there are either very specific mechanisms how those variants functionally transduce cells and/or that the different models have strong biological similarities. The latter would indicate that the retinal organoids and iRPE could serve as predictive models of primary human retina cells. In contrast, it became apparent that the capsids that highly transduce human retinal cells were very inefficient at transducing the murine retina and vice-versa. The only two exceptions for this trend were AAV-7m8, which was highly efficient in almost every model, and to a lesser degree AAV2-M1, which also showed a moderate level of transduction in every model assessed. Another interesting observation was the different performance of AAV2-M4 in mature and immature iRPE. A possible explanation could be related to the utilization of a cellular receptor or co-receptor expressed by more mature iRPEs, but not by immature iRPEs. These findings highlight the importance of selecting novel AAVs in the most relevant models possible.

Interestingly, AAV-7m8 was not only the strongest transducer in all human retina models, but also had a high RNA-to-DNA ratio in the NGS results, indicating a very efficient intracellular trafficking profile previously observed in vitro^14^. AAV2-M4 shows a similar pattern in human retinal explants and human retinal organoids, but shows the opposite trend in the RPE models. This would indicate that AAV2-M4 also has a very efficient intracellular trafficking properties, but they may be limited to certain preclinical models and cell types.

For benchmarking the transduction performance, despite the fact that novel AAV variants that outperform AAV-7m8 in several model systems have recently been published^5–8^, we decided not to include them in our final comparison studies. This decision was based on the fact that our variants did not outperform the AAV-7m8 in most of our studies. Instead, we focused on investigating how well different AAV variants compared in the individual model systems, with the goal to understand both the limitations of the AAV capsids as well as the limitation of each model system.

Our results support the view, that no model should be used in isolation, as each presents a unique set of biases. This was most visible for the two-round selection where the ‘free-floating’ explants would have been of low value without the subsequent selection in polarized explant culture. Additionally, while the primary human tissue is undoubtedly a good model for human retina cells, the comparatively short culture periods are a limitation for the interpretation of the results. Conducting the same experiments for extended culture periods in retinal organoids provided us with an insight that the vectors would probably perform in a similar way in preclinical models that enable long-term functional evaluations.

To our knowledge, this publication was the first to use retinal explant cultures as a model system for directed evolution of novel AAV variants. Some of the novel highly performing capsids identified in our studies were able to transduce several human models of retinal cells and show promising performance in specific cell types. However, as already mentioned above, one of the limitations of this approach was that the human retinal explants have a relatively short life in culture, which does not recapitulate correctly physiological conditions in vivo, both in animal models and human patients. Retinal explant cultures are also generally from older donors, making it difficult to assess whether ‘younger’ retinas would react to the vectors in the same way. Using retinal organoids in parallel to retinal explants can help with both limitations as they can be cultured for longer periods of time and based on their differentiation stage are probably better models of ‘younger’ retinas compared to post-mortem explants.

Furthermore, our selection approach did not enable the selection for AAV variants with tropism towards specific cell types. Similar limitation applied also to the NGS-based validation studies. However, both caveats can be addressed in future studies with the use of tissue-restrictive promoters, such as the hSYNrv-p40 promoter for glia detargeting capsid selection (Supplementary Fig. 4) or a photoreceptor-restrictive promoter in NGS validation studies.

To better address the in vivo translatability, future AAV directed evolution studies targeting human retina could incorporate cross-species alternative rounds of selections in in vivo non-human primate model and ex vivo human retina model. This could lead to the identification of highly potent capsid variants but would come at a higher cost and unique logistical challenges associated with the use of NHPs, not to mention critical ethical considerations related to the use of NHP in biomedical research.

Overall, our data support the hypothesis that AAV2-M4 may be a superior capsid for the transduction of Müller glia cells in the primary human retina following IVT injection. While many gene therapy approaches aim at transducing the light-sensing photoreceptor cells, targeting the Müller glia cells was recently shown as an approach to treat retinal dystrophies caused by mutations in *CRB1*^22^. Thus this new variant has a potential to play an important role for development of gene therapies that do not require targeting the photoreceptors and/or depend on expression of secreted proteins.

## Materials and methods

### Generation of the ‘AAV Kit’

In a small initial study, the previously published 30 ‘AAV Kit’ was used^14^. Afterwards, fifty-one AAV variants were chosen to generate a second-generation ‘AAV Kit’ (Supplementary Table 1). The equimolar mix of individually produced, barcoded AAV variants was performed as previously published^14^. In addition to extending the ‘AAV Kit’ by 21 variants, all capsids were packaging a set of ssAAV-cytomegalovirus (CMV) promoter-enhanced green fluorescent protein (eGFP)-six nucleotide barcode-woodchuck hepatitis virus post-transcriptional regulatory element-bovine growth hormone poly-adenylation signal. This set was a 1:1 mix of two BCs that were unique to each AAV variant.

All variants were produced by linear polyethyleneimine hydrochloride (40,000, PEI MAX, Polysciences, Cat# 49553-93-7) transfection of human embryonic kidney (HEK) 293 T cells (ATCC, Cat# CRL-3216) and subsequently purified using iodixanol gradient centrifugation as previously published^14^.

### Generation of the peptide insertion library

An AAV2-based library with a seven amino acid insertion into the variable region VIII was used in this study as the starting point for the directed evolution. The library (in the FT platform) was identical to an AAV2 library used in a previously published directed evolution study in primary human hepatocytes^13^.

### Cloning of the novel capsid peptide variants

Based on NGS results, novel capsids for the human retina were generated using a PCR reaction with the respective primers for each novel variant carrying the peptide sequence as overhangs (Supplementary Table 2). The PCR template was always pRep2Cap-lco2-SfiI. The forward primers and the reverse primers each inserted half of the peptide sequence as the 5’-prime overhang of the primer. The 3’ end was identical as the template binding region. After the PCR product was gel purified (Zymogen, Cat# D4001), the plasmids were circularized using KLD enzyme mix (New England Biolabs, Cat# M0554S) which generated the complete peptide sequence, transformed, and plated on LB-agar plates containing ampicillin.

All novel and control variants were produced with double barcodes as described above by PEI MAX transfection of HEK293T cells and subsequently purified using iodixanol gradient centrifugation as previously published^14^. A ‘Retina AAV Kit’ was generated with these variants using previously published protocols^14^.

For the ‘Retina AAV Kit version 2’ that was used for mouse studies, the transgene carried mCherry rather than eGFP. The generation and production of the ‘Retina AAV Kit version 2’ were identical to the ‘Retina AAV Kit’, but with three barcodes per AAV variant rather than two.

### Vector quantification

2µL aliquots of the produced vector preps and libraries were incubated at 94 ℃ with 49 µL alkaline digestion buffer (25 mM NaOH, 0.2 mM EDTA) for 10 min. Following the incubation, the pH was neutralized with 49 µL of neutralization buffer (40 mM Tris–HCl, pH 5.0, 0.05% Tween20). The samples were further diluted with the 1:100,000, 1:500,000, and 1:2,000,000 dilutions quantified by droplet digital PCR (ddPCR) using the EvaGreen Supermix (BioRad, Cat# 1864036) following the manufacturer’s instructions. Vectors and libraries were quantified using primers for the eGFP gene (F: 5′-GAGGTGAAGTTCGAGGGC-3′; R: 5′-CTTGTGCCCCAGGATGTTG-3′) at a final concentration of 75 nM^23^. The final titer was established by averaging the results from the three dilutions.

### Illumina-based amplicon sequencing

NGS was performed as previously described for the AAV Kit (barcode NGS)^14^ and the peptide insertion library^13^. In brief, following DNA/RNA extraction using the AllPrep Mini Kit (QIAGEN, Cat# 80204), RNA was used for first-strand cDNA synthesis with the SSIV Kit (Invitrogen, Cat# 18091050). Both DNA and cDNA were amplified with the following primers for generation of a PCR amplicon that was submitted for paired-end-150 Illumina amplicon seq:

- 30/51 and retina AAV Kit (excluding the version 2 kit for mouse studies)
  - Forward: GCTGGAGTTCGTGACCGCCG
  - Reverse: CAACATAGTTAAGAATACCAGTCAATCTTTCAC
- Retina AAV Kit version 2 for mouse studies
  - Retina AAV Kit version 2 for mouse studies
  - Forward: GCCTCTTCCGCGTCTTCG
  - Reverse: ATGGCTGGCAACTAGAAGGC
- Recovery of peptide variants from library
  - Forward: CTAACCCTGTGGCCACGG
  - Reverse: CGTCTCTGTCTTGCCACACC

Each sample had a minimum read depth of 1,000,000 reads. The NGS results from all samples and produced libraries and mixes pre-treatment were analyzed using a previously published custom script^24^. The results from DNA and cDNA samples were normalized to the vectorized input mix to account for imperfection in the equimolar mix of variants as previously described^14^.

### Human retina explant isolation

Human retinal explant and human RPE culture studies were performed with approval from the Sydney Children’s Hospitals Network Human Research Ethics Committee (HREC/17/SCHN323) and the New South Wales Organ and Tissue Donation Service, following corneal removal for tissue transplantation. The New South Wales Organ and Tissue Donation Service has obtained informed consent from all subjects and/or their legal guardian. All methods were performed in accordance with the relevant guidelines and regulations. The retina was dissected from the eye and placed in sterile Ames’ medium before further culture/use. Ames’ medium was prepared according to manufacturers’ protocol, then aerated with carbogen (95% O_2_/5% CO_2_). To 1 L medium, the following was added: 10 mL penicillin/ streptomycin solution (100 U/ml penicillin, 100 µg/ml streptomycin mixed solution); 10 mL L-glutamine (0.292 mg/ml); and 5 mL horse serum (0.5%). Medium was aerated with carbogen until use. The initial medium was replaced with ~ 30 mL Ames’ medium in sterile specimen jars containing donor eyes. The whole retina was transferred into carboxygenated medium at room temperature (Supplementary Fig. 7a,b). The retina was separated into quadrants and each quadrant separated into a central and peripheral portion and cultured on separate membranes (Supplementary Fig. 7c). Each piece of retina was approximately 1 × 2 cm, rectangular in shape, to ensure optimal cell numbers during cell sorting process. Alternatively, 5 to 8 mm trephines (using a biopsy punch) were taken from areas of interest (macula, periphery, mid-periphery, Supplementary Fig. 7d)^15^.

### Free-floating retinal explant immersion method

During Round 1 selection (described below) the “Free-floating Retinal Explant Immersion method” of culture was used. Human retinal explants were obtained as described above, with 5- and 8-mm trephines taken from areas of interest (macular, mid-periphery, periphery). The smaller pieces were taken as controls (negative and positive). Larger pieces were used for carrying out first round AAV library selection. The retinal explants (5 and 8 mm) were incubated as free-floating tissue pieces in 1 mL of Ames media (Sigma-Aldrich, Cat# A1420-10X1L) in 24-well plate for 16 h, 37 °C, 5% CO_2_.

### Interphase retinal explant culture system (polarized explants)

Donor eyes were obtained and prepared as retinal explant cultures as described above, with trephines taken from areas of interest (macular, mid-periphery, periphery). During Round 2 selection (described below) the “Interphase Retinal Explant Culture System” was used (Retinal ganglion cell side up, photoreceptors down), to simulate intravitreal injection and with the aim to select for photoreceptors (adapted from^25^). Four retinal tissue pieces, each of approximately 1 × 2 cm^2^ in size, were dissected out and placed onto the prepared tissue culture insert for second round AAV library selection. In addition, 8 mm trephine pieces were taken for use as control stains (negative and positive). A tissue culture insert (0.4 µm pore size, 30 mm diameter; Merck-Millipore) was prepared for each retinal piece. The insert was filled with media then the retinal piece was transferred onto the insert, ganglion cell side up. A pipette was used to remove the media from inside the membrane.

A gentle suction was applied (for ~ 30 s) to the underside of the membrane to allow attachment of the tissue. This was done by placing a piece of filter paper underneath the membrane, and the retina was kept on membrane. Cell culture dishes (100 mm diameter, 20 mm depth) were filled with 65 ml of Ames’ medium (containing 0.192% sodium bicarbonate, 100 U/mL penicillin, 100 mg/mL streptomycin, 0.5% Horse Serum, and 0.292 mg/mL l-glutamine) and the tissue culture insert rested on the custom printed filter stands (polylactic acid polymer using a 3D-printer by Dr. Suat Dervish at the Westmead Institute of Medical Research, Sydney, Australia) and then placed into the cell culture dish, ensuring the retina was contacted with the medium via the filter on the photoreceptor side and with the incubator atmosphere (5% CO_2_, 95% air, 37 °C, humidified) on the ganglion cell side. The photoreceptor side of the retinal explant faced down towards the media, while the ganglion cell layer side faced the top and was exposed to air. These were used for carrying out the second round AAV library selection and further validation. Retinal explants were incubated as larger tissue pieces (1 × 2 cm^2^) on the well insert and stand, and the free-floating tissue pieces (8 mm) in 1 mL of Ames media in 24-well plate, for 16 h, 37 °C, 5% CO_2_.

### Library selection—round 1

Round 1 selection of the FT-SFFV-GFP.AAVLib2^NNK7^ library (same library as previously published)^13^ was performed on primary human retina explants (Free-floating Retinal Explants). The double CsCl purified library was added to retina explants floating in media (Free-floating Retinal Explant Immersion method). The entire culture period was capped at five days to ensure integrity of the post-mortem tissue.

The library was added at varying volumes (5, 10, 20 and 25 µL [corresponding to doses: 6.5 × 10^10^, 1.3 × 10^11^, 2.6 × 10^11^ and 3.3 × 10^11^ vector genomes (vg) per well, respectively]) to each of the 8 mm retinal explants (× 4) and incubated for further 4 days, at 37 °C, 5% CO_2_. Media change was performed every 24 h (Selection Round 1). For one of the 8 mm retinal explants, AAV5.CMV-eGFP (5.10 × 10^10^ vg per well) was added as a single stain eGFP control. Two other 5 mm retinal explants were used as unstained and CD73-allophycocyanin (APC) single stain controls. Cells were incubated for 4 days, at 37 °C and 5% CO_2_, with media changed every 24 h.

Media was removed from each well and the retinal explants transferred to a petri dish with minimal Ames/DMEM medium. Retinal explants were chopped into fine pieces with a scalpel before being transferred to a 1.5 mL Eppendorf tube with 500 µL digestion buffer (2.5 mg/mL papain, 0.1 mg/mL DNase, 1.5 µg/mL superoxide dismutase in HBSS with 5 mM MgCl_2_) and digested for 5 min at 37 °C. The temperature was reduced to 8 °C and maintained for 20 min before the tube was centrifuged at 300 × g for 5 min at 4 °C and supernatant removed. The pellet was resuspended in 500 µL neutralization buffer (50 µg/mL antipapain, 0.1 mg/mL DNase, 1.5 µg/mL superoxide dismutase in HBSS with 5 mM MgCl_2_) and incubated for 10 min on ice. Aliquots of 50–100 µL were taken for negative and single stain controls at this time. Samples were centrifuged at 300 × g for 5 min at 4 °C and the supernatant removed and discarded.

To stain cells with CD73-APC conjugate antibody (1:10), the pellet was resuspended in staining buffer (10 mg/mL bovine serum albumin, 0.1 mg/mL DNase, in PBS with 5 mM MgCl_2_) containing the antibody and incubated at 4 °C for 40 min. For unstained controls, the pellet was resuspended and incubated at 4 °C for 40 min. For FACS analysis, cells were again centrifuged, washed in PBS, and then resuspended in FACS buffer (2% FBS, 0.1 mg/mL DNase, in PBS with 5 mM MgCl_2_, 0.22 µm filtered). The stained cell suspension was then filtered through a 70 µm mesh into a FACS tube.

A GFP^+^/CD73^+^ population from each of the four conditions (i.e., each of the different amounts of library added to the explants) was identified. RNA and DNA was extracted, cDNA produced and processed for NGS.

### Library selection—round 2

The secondary library (enriched FT-SFFV-GFP.AAVLib2^NNK7^ library from RNA/cDNA amplicons combined from all conditions of the first round of selection) was produced and packaged as described above but purified using iodixanol gradient ultracentrifugation^13^. The secondary library was added at varying doses (7.4 × 10^10^, 1.5 × 10^11^, 3.0 × 10^11^ and 6.0 × 10^11^ vg per well, corresponding to 12, 25, 50, and 100 µL of library prep) to each of the larger tissue pieces (1 × 2 cm^2^), on top of the ganglion cell layer side, and incubated for 4 days, 37 °C, 5% CO_2_, with Ames’ media change every 24 h. The entire culture period was limited to five days to ensure integrity of the post-mortem tissue.

For the 8 mm retinal explants, AAV-7m8.CMV-eGFP (1.0 × 10^10^ vg per well) was added for a single stain eGFP control. There were two other conditions for the 8 mm retinal explants: unstained control and CD73-APC single stain control. The tissue was incubated for further 4 days, at 37 °C, 5% CO_2_, with a media change every 24 h.

Four days post transduction, tissues were harvested, dissociated, labelled for CD73 surface receptor (photoreceptor marker in mature photoreceptors), and analyzed for eGFP expression. CD73-positive cells were sorted for medium and high dose conditions (M/H) and 5% GFP positive CD73 cells were detected in those conditions. Cells transduced with the lowest dose of AAV (L) were not sorted. RNA was extracted from non-sorted (L) or CD73-positive cells (M/H) using trizol-isopropanol precipitation and processed for cDNA synthesis as described previously and cDNA was amplified for NGS analysis^13,14^.

Retina explant transductions for immunofluorescent analysis were performed with 8-mm hole puncher as interphase cultures in trans wells. A dose of 5.0 × 10^10^ vg per well of AAV-7m8.CMV-eGFP or AAV2-M4.CMV-eGFP was added. The tissue was incubated for further 4 days, at 37 °C, 5% CO_2_, with a media change every 24 h prior to fixing and preparation for immunofluorescence. Tissue integrity after 5 days of culture can be seen in Supplementary Fig. 7e.

### Maintenance of stem cells

Human stem cell work work was approved by the Children’s Medical Research Institute (NL 21.04). All methods were performed in accordance with the relevant guidelines and regulations. The hiPSC line (UCLOOi017-A-1) and hESC H9 (WiCell) were maintained on feeder-free conditions on Essential 8 media (Gibco, Cat# A1517001) and Geltrex (Gibco, Cat# A1413202) coated 6-well plates. When 70% confluent, these cells were dissociated using Versene solution (Gibco, Cat# 15040066) at 37 °C for 5 to 10 min. The dissociated clumps were washed once in PBS with centrifugation at 900 rpm for 5 min. The resulting cell pellet was further dissociated and resuspended in 1 ml E8 media and between 70 and 100ul of cells was added to each well of the 6-well plate containing 2 ml E8 and 10 µM ROCK inhibitor (Y-27632 dihydrochloride, Tocris) for 24 h. Daily feeding with E8 was continued for further maintenance culture.

### Retinal organoid differentiation culture

To generate retinal organoids, hiPSCs were maintained as described above until 90–95% confluent. Essential 6 media (Gibco, Cat# A1516401) was added to the cultures for two days (replaced fresh between day 1 and 2 of differentiation). At day three of differentiation, media was replaced with a pro-neural induction media (PIM, composed of Advanced DMEM/F12, N2 supplement, L-Glut, non-essential amino acid and Antibiotic–antimycotic). Neuroretinal vesicles started to appear between weeks four and seven of culture. They were discerned by the typical transparent neural epithelium that arises from islands of pigmented RPE cells. During this period, they were manually excised with 19G needles and kept individually in low binding 96-well plates (Nunclon sphere, ThermoFisher) to mature into retinal organoids and maintained in retinal differentiation media (RDM composed of DMEM, F12 Nutrient mix, B27 without retinoic acid, Antibiotic–antimycotic). At six weeks of differentiation, media was replaced with RDMF (RDM supplemented with 10% FBS, 100 µM taurine [Sigma, Cat# T4871] and 2 mM glutamax). At ten weeks, RDMF was replaced by ALT70 media (composed of Advanced DMEM/F12 supplemented with all the components of RDMF and 1 µM retinoic acid,^26^) and retinal organoids were transferred to low binding 24-well plates. At twelve weeks of differentiation, ALT70 media was replaced with ALT90 media (Alt70 media supplemented with 1% N2 supplement and reduced retinoic acid concentration of 0.5 µM,^26^). All media described here were replaced every other day.

### AAV transduction of retinal organoids in vitro

All organoids were 120 days old at the time of AAV exposure. AAV vectors (2 × 10^10^ vg/organoid) were added to a volume of 300 µL using fresh ALT90 media used to culture the retinal organoids. The organoids (2–4) were then transferred to low binding 24 well plates (Costar, Corning) and media was completely replaced with ALT90 containing the AAV vectors. Organoids were incubated at 37 °C for half a day before adding another 700 µL of fresh media. After overnight incubation at 37 °C, an additional 1 mL of ALT90 media was added to retinal organoids. After 48 h at 37 °C, organoids were fed every other day. Organoids were harvested for NGS preparation, immunofluorescence, or flow cytometry 14 days after AAV exposure.

### RPE cultures

Differentiated RPE cultures derived from hiPSCs were generated as previously described^12^ from the line UCLOOi017-A-1^11^. Culture conditions and protocols were previously described^12^. Human iPSC work was approved by the Children’s Medical Research Institute (NL 21.04). All methods were performed in accordance with the relevant guidelines and regulations. The flow cytometry was performed by dissociating and washing the cells. The cells were resuspended in PBS containing 10% FBS and 5 mM EDTA and 1 µg/mL DAPI. The flow cytometry was performed on a BD LSR Fortessa instrument. To be able to detect two distinct morphologically cell populations, exclusion of DAPI-positive cells was performed first. Only then were the two populations identified based on forward scatter and side scatter intensity (Supplementary Fig. 5a).

Primary RPE cultures were generated from donor *post-mortem* human eyes, as previously described^27^, aged between 45 and 65 years old (*post-mortem* delay < 16 h), Briefly, the lens/iris was excised, and the retina separated from the RPE and removed from the eyecup. The RPE-lined eyecup was filled with 0.25% trypsin/0.1% EDTA, 37 °C for 40–60 min to isolate the RPE in single cell suspension; after pelleting, the RPE cells were cultured in 35 mm tissue culture dishes in DMEM/20% FBS at 37 °C in 5% CO_2_. Cultures after passage (P) 0 were grown in DMEM/10% FBS. Media was changed weekly, and cultures were passaged with 0.25% trypsin/0.1% EDTA; experiments were conducted on P1 cultures.

Human RPE cells were seeded at 2 × 10^5^ cells/mL, in DMEM/10% FBS, prior to incubation with either 51 ‘AAV Kit’ or ‘Retina AAV Kit’ at 10,000 MOI, for 3 or 7 days. Following the end of the incubation period, the RPE cells were isolated with 0.25% trypsin/0.1% EDTA, pelleted, and the DNA and RNA was extracted for NGS. RPE cells with no AAV was included as a control.

### AAV transduction of mouse retina in vivo

All animal use was approved by the Institutional Animal Ethics Committee of the Harry Perkins Medical Research Institute (AE0198) and was in accordance with the Australian Code for the Care and Use of Animals for Scientific Purposes and the ARVO Statement for the Use of Animals in Ophthalmic and Vision Research. All mice were group-housed in a climate-controlled facility on a 12-h light/dark cycle with food and water ad libitum. All animal studies were performed in accordance with the ARRIVE guidelines and in accordance with the relevant guidelines and regulations.

### Ocular injections

Six wildtype Chrnb4.GFP mice^28,29^ (4–6 weeks old) were anesthetised with an intraperitoneal injection of 80 mg/kg Ketamine (Ceva Animal Health Pty Ltd) and 10 mg/kg Ilium Xylazil-100 (Troy Laboratories), and pupils dilated with a drop of 1% Tropicamide (Alcon, Fort Worth, USA) applied on the corneal surface. The AAV vector library was delivered using the UltraMicro Pump (UMP3) and a NanoFil 10µl syringe (World Precision Instruments) and three animals received subretinal injections in both eyes and three animals received intravitreal injections in both eyes. For the subretinal injection, surgical scissors are used to gently cut through the connective tissue surrounding the injection site. A small scleral incision is done using the tip of a disposable bevelled 26-gauge needle at the temporal side of the optic nerve. A blunt 35-gauge needle attached to the pump is inserted into the sclera and when the tip of the needle reaches the nasal subretinal space, the required 1µl volume is injected. To confirm if the subretinal injection was successful, detachment in the inferior nasal (if the left eye is injected) and the superior nasal (if the right eye is injected) subretinal space is detected by OCT imaging using a Bioptigen OCT system straight after the subretinal injections. For intravitreal injections, a small cut is made on the limbus area with a bevelled 29-gauge needle. A blunt 35-gauge needle attached to the NanoFil syringe is then inserted into the eye carefully to avoid injury to the lens. Once the tip of the needle reaches the vitreous at the back of the eye, 1µl volume is injected.

### Tissue collection and DNA/RNA extraction

At 28 days post-injection, animals were euthanised via cervical dislocation and the retinas were extracted for either flow associated cell sorting (FACS, n = 3 eyes/injection type) or whole retina extraction (n = 3 eyes/injections type). Retinal dissociation and FACS of cones and non-cones cells were done as previously described^30^. DNA/RNA extraction were done using the Qiagen All Prep DNA/RNA kit (Qiagen, USA) and followed manufacturer’s instructions.

### Neutralization assay and HeLa cell transduction

HeLa cells were seeded at density of 150,000 cells per well in 24-well plate 16 h prior to AAV exposure. For the HeLa cell baseline performance for each capsid, AAV2, AAV2-7m8, AAV2-1.3, AAV2-L1, AAV2-L2, AAV2-M1, and AAV2-M4 were added at doses of 5000 vg/cell and 500 vg/cell. Media was changed 6 h after AAV exposure. Cells were kept in culture for an additional 42 h before preparation for flow cytometry. eGFP fluorescence was quantified using a BD LSR Fortessa instrument. Data was analyzed using FlowJo 10. For the neutralization assay, the conditions of HeLa cell cultures were the same as outlined above, but the vector preps were adjusted to have the same final volume required for transduction and incubated with undiluted (neat), 1:4 diluted, and 1:16 diluted IVIg. Transduction was performed in triplicates and the vector performance in the presence of IVIg was normalized to the vector performance in absence of IVIg performed in parallel. eGFP fluorescence was established using a BD LSR Fortessa instrument. Data was analyzed using FlowJo 10.

### Data analysis, statistical analysis, and visualization

NGS data was analyzed using custom python scripts and workflows integrated in the Geneious software as previously published^13,14,31^. All data was analyzed using Microsoft Excel and GraphPad Prism. Statistical analysis was performed in GraphPad Prism using the recommended setting for the respective dataset. Graphs and heatmaps were generated using GraphPad Prism. Data visualization was aided by the generation of schematics and workflows using Adobe Illustrator.

### Supplementary Information

Supplementary Information.

## Data Availability

The sequencing datasets generating novel capsid sequences developed during the current study are available on Genbank, accession numbers OR837062-OR837081. All other datasets used or analyzed during the current study are available from the corresponding author on reasonable request.
