# Supplementary material for: AAV capsid bioengineering in primary human retina models
Source: Sci Rep. 2023 Dec 11;13:21946. doi: 10.1038/s41598-023-49112-2 (PMC10713676; doi:10.1038/s41598-023-49112-2)
Supplement: Supplementary file 1 — Supplementary Information. [file 41598_2023_49112_MOESM1_ESM.docx]

Supplemental information

# Supplementary Table 1. AAV variants in 51 ‘AAV Kit’

| **#** | **Capsid variant** | **Origin** | **Library type (if directed evolution)** | **Citation** |
| --- | --- | --- | --- | --- |
| 1 | AAV1 | Human cell culture isolate | N/A | 1 |
| 2 | AAV2 | Human cell culture isolate | N/A | 2, 3 |
| 3 | AAV3b | Human cell culture isolate | N/A | 2 |
| 4 | AAV4 | Non-human primate cell culture isolate | N/A | 4 |
| 5 | AAV5 | Human cancer isolate | N/A | 5 |
| 6 | AAV6 | Human cell culture isolate | N/A | 3 |
| 7 | AAV7 | Non-human primate isolate | N/A | 6 |
| 8 | AAV8 | Non-human primate isolate | N/A | 6 |
| 9 | AAV9 | Human isolate | N/A | 7 |
| 10 | AAV10 | Non-human primate isolate | N/A | 8 |
| 11 | AAV11 | Non-human primate isolate | N/A | 8 |
| 12 | AAV12 | Non-human primate isolate | N/A | 9 |
| 13 | AAV13 | Human cell culture isolate | N/A | 10 |
| 14 | AAV-rh10 | Non-human primate isolate | N/A | 11 |
| 15 | AAV-h.Lvr6 | Human liver isolate | N/A | 12 |
| 16 | AAV-2i8 | Murine muscle rational design | N/A | 13 |
| 17 | AAV-Anc80 | Ancestral reconstruction | N/A | 14 |
| 18 | AAV-DJ | HuH-7 cells directed evolution | Shuffled library | 15 |
| 19 | AAV-DJ8 | AAV-DJ modification for murine tropism | Shuffled library + rational design | 15 |
| 20 | AAV-LK01 | Human liver xenograft directed evolution | Shuffled library | 16 |
| 21 | AAV-LK02 | Human liver xenograft directed evolution | Shuffled library | 16 |
| 22 | AAV-LK03 | Human liver xenograft directed evolution | Shuffled library | 16 |
| 23 | AAV-LK19 | Human liver xenograft directed evolution | Shuffled library | 16 |
| 24 | AAV-NP6 | Human muscle xenograft directed evolution | Shuffled library | 17 |
| 25 | AAV-NP22 | Human muscle xenograft directed evolution | Shuffled library | 17 |
| 26 | AAV-NP40 | Human liver xenograft directed evolution | Shuffled library | 18 |
| 27 | AAV-NP59 | Human liver xenograft directed evolution | Shuffled library | 18 |
| 28 | AAV-NP66 | Human muscle xenograft directed evolution | Shuffled library | 17 |
| 29 | AAV-NP84 | Human liver xenograft directed evolution | Shuffled library | 18 |
| 30 | AAV-NP94 | Human muscle xenograft directed evolution | Shuffled library | 17 |
| 31 | AAV-KP1 | Human islet directed evolution | Shuffled library | 19 |
| 32 | AAV-KP2 | Human islet directed evolution | Shuffled library | 19 |
| 33 | AAV-KP3 | Human islet directed evolution | Shuffled library | 19 |
| 34 | AAV-7m8 | Murine retina directed evolution | Peptide insertion | 20 |
| 35 | AAV-PHP.eB | Murine CNS directed evolution | Peptide insertion | 21 |
| 36 | AAV2-Retro | Murine CNS directed evolution | Peptide insertion | 22 |
| 37 | AAV-HRP5 | HuH-7 cells directed evolution | Shuffled library | 23* |
| 38 | AAV-HRS1 | HuH-7 cells directed evolution | Shuffled library | 23* |
| 39 | AAV-HRS19 | HuH-7 cells directed evolution | Shuffled library | 23* |
| 40 | AAV-CD15 | Human HSPC directed evolution | Shuffled library | 23* |
| 41 | AAV-CD-SYD01 | Human HSPC directed evolution | Shuffled library | 23* |
| 42 | AAV-CD-SYD03 | Human HSPC directed evolution | Shuffled library | 23* |
| 43 | AAV-CD-SYD09 | Human HSPC directed evolution | Shuffled library | 23* |
| 44 | AAV-T33 | Human T-cell directed evolution | Shuffled library | 23* |
| 45 | AAV-R588I | Human liver xenograft replicative evolution | Shuffled library | 12 |
| 46 | AAV-N496D | Human liver xenograft directed evolution | Shuffled library | 24 |
| 47 | AAV-SYD04 | Human liver xenograft directed evolution | Shuffled library | 25 |
| 48 | AAV-SYD11 | Human liver xenograft directed evolution | Shuffled library | 25 |
| 49 | AAV-SYD12 | Human liver xenograft directed evolution | Shuffled library | 25 |
| 50 | AAV2-RC01 | Human liver xenograft directed evolution | Peptide insertion | 26 |
| 51 | AAV2-RC02 | Human liver xenograft directed evolution | Peptide insertion | 26 |

*variants 37-44 were previously published in a PhD Thesis.^23^

# Supplementary Table 2. Oligonucleotides for peptide cloning

| 1.1_F | AGGCGGCGCACTCTGGCCCTGGAGGTTGGT |
| --- | --- |
| 1.1_R | CGGCATCCGTCTGAGGCGGCCGCGACCGCA |
| 1.2_F | CTGCGTAGTCCTCTGGCCCTGGAGGTTGGT |
| 1.2_R | TTTCATCCGCCGGAGGCGGCCGCGACCGCA |
| 1.3_F | CTGCTTAGGCCTCTGGCCCTGGAGGTTGGT |
| 1.3_R | CCTACGCAGCCTAAGGCGGCCGCGACCGCA |
| 1.4_F | ATAAGAAGGCCTCTGGCCCTGGAGGTTGGT |
| 1.4_R | AGTCCTTCGAATCAGGCGGCCGCGACCGCA |
| 1.5_F | CACAACACTACTCTGGCCCTGGAGGTTGGT |
| 1.5_R | AGTTCGCGTTGTGAGGCGGCCGCGACCGCA |
| L1_F | AGGCTGCTGCCTCTGGCCCTGGAGGTTGGT |
| L1_R | CAGAATACTCGTCAGGCGGCCGCGACCGCA |
| L2_F | CTGAAGCGTACTCTGGCCCTGGAGGTTGGT |
| L2_R | CGGACGATGGCGAAGGCGGCCGCGACCGCA |
| L3_F | AGCCAGATGCCTCTGGCCCTGGAGGTTGGT |
| L3_R | GTGGCGCCGCCGCAGGCGGCCGCGACCGCA |
| L4_F | ACGCCAAGGCCTCTGGCCCTGGAGGTTGGT |
| L4_R | GAGTCGTCTCAGGAGGCGGCCGCGACCGCA |
| L5_F | CGTAGTCGTACTCTGGCCCTGGAGGTTGGT |
| L5_R | CGTGATATGCCTAAGGCGGCCGCGACCGCA |
| M1_F | CCTCTGCCGCCTCTGGCCCTGGAGGTTGGT |
| M1_R | ATTCCGGGGGGGGAGGCGGCCGCGACCGCA |
| M2_F | ATGATGATTACTCTGGCCCTGGAGGTTGGT |
| M2_R | ACGAATAATCCGAAGGCGGCCGCGACCGCA |
| M3_F | ACCATAATTCCTCTGGCCCTGGAGGTTGGT |
| M3_R | AGGCAGGATTCTCAGGCGGCCGCGACCGCA |
| M4_F | CCGAGGCAAACTCTGGCCCTGGAGGTTGGT |
| M4_R | CGGGATGCTCCTAAGGCGGCCGCGACCGCA |
| M5_F | CTTATCCAGACTCTGGCCCTGGAGGTTGGT |
| M5_R | AAGAATGCGACGAAGGCGGCCGCGACCGCA |
| H1_F | CTTATTCTTCCTCTGGCCCTGGAGGTTGGT |
| H1_R | GATACGCCGGTGAAGGCGGCCGCGACCGCA |
| H2_F | ATTCTGATTCCTCTGGCCCTGGAGGTTGGT |
| H2_R | GAGACTAAGAGGCAGGCGGCCGCGACCGCA |
| H3_F | AAGCTGCTTACTCTGGCCCTGGAGGTTGGT |
| H3_R | CCGACTAATAATAAGGCGGCCGCGACCGCA |
| H4_F | CGGATTCTGCCTCTGGCCCTGGAGGTTGGT |
| H4_R | AAGCTGGGTTCGGAGGCGGCCGCGACCGCA |
| H5_F | ATTAATCTTACTCTGGCCCTGGAGGTTGGT |
| H5_R | CCGAATGCTTCGAAGGCGGCCGCGACCGCA |

# Supplementary Table 3. Selected peptides

| **Variant name** | **Peptide** | **Counts** | **Percentage** |
| --- | --- | --- | --- |
| Round 1 | | | |
| **1.1** | SAPPRHPSE | 25658 | 1.50% |
| **1.2** | RTTQFHPPE | 19916 | 1.17% |
| **1.3** | RPKQPTQPK | 13524 | 0.79% |
| **1.4** | RPSYSPSNQ | 13341 | 0.78% |
| **1.5** | SSVVSSRCE | 13163 | 0.77% |
| Round 2 – Unsorted; low dose | | | |
| **L1** | RKNKDTPVK | 51022 | 2.21% |
| **L2** | RNQNETKRQ | 41336 | 1.79% |
| **L3** | SKQLPTNNK | 39001 | 1.69% |
| **L4** | RQNPKLGSE | 33414 | 1.44% |
| **L5** | SKINPNASK | 27632 | 1.19% |
| *M4* | *SLPRRDAPK* | *27304* | *1.18%* |
| Round 2 – CD73^+^; medium dose | | | |
| *L1* | *RKNKDTPVK* | *89900* | *2.94%* |
| **M1** | RRQRIPGGE | 86102 | 2.81% |
| **M2** | SNHHTNNPK | 78538 | 2.56% |
| **M3** | RNYGRQDSQ | 71595 | 2.34% |
| **M4** | SLPRRDAPK | 69491 | 2.27% |
| **M5** | SLDKKNATK | 69108 | 2.26% |
| *L2* | *RNQNETKRQ* | *60017* | *1.96%* |
| *L3* | *SKQLPTNNK* | *50421* | *1.65%* |
| M6 | SSQRLPTTQ | 49495 | 1.62% |
| M7 | RPTKHLDRE | 48752 | 1.59% |
| *H6* | *RRKAENQMK* | *45759* | *1.49%* |
| *L5* | *SKINPNASK* | *41970* | *1.37%* |
| Round 2 – CD73^+^; high dose | | | |
| **H1** | RQQPQNTRQ | 228671 | 10.29% |
| **H2** | STLQRTMAK | 128948 | 5.80% |
| **H3** | RHLAVAPPQ | 127910 | 5.75% |
| **H4** | RPWRESSQE | 126922 | 5.71% |
| **H5** | STTTRDMPK | 126428 | 5.69% |
| H6 | RRKAENQMK | 119396 | 5.37% |
| H7 | RRGSDPVRK | 118018 | 5.31% |
| H9 | RKKNEETKK | 113619 | 5.11% |
| H11 | RRINMATGQ | 111675 | 5.02% |
| *L1* | *RKNKDTPVK* | *107193* | *4.82%* |

# Supplementary Table 4. Transgene sequences used in the study

| ssAAV-ITR-CMV-eGFP-BC-WPRE-BGHpA-ITR | TTGGCCACTCCCTCTCTGCGCGCTCGCTCGCTCACTGAGGCCGGGCGACCAAAGGTCGCCCGACGCCCGGGCTTTGCCCGGGCGGCCTCAGTGAGCGAGCGAGCGCGCAGAGAGGGAGTGGCCAACTCCATCACTAGGGGTTCCTGGTACCGAGGACATTGATTATTGACTAGTTATTAATAGTAATCAATTACGGGGTCATTAGTTCATAGCCCATATATGGAGTTCCGCGTTACATAACTTACGGTAAATGGCCCGCCTGGCTGACCGCCCAACGACCCCCGCCCATTGACGTCAATAATGACGTATGTTCCCATAGTAACGCCAATAGGGACTTTCCATTGACGTCAATGGGTGGAGTATTTACGGTAAACTGCCCACTTGGCAGTACATCAAGTGTATCATATGCCAAGTACGCCCCCTATTGACGTCAATGACGGTAAATGGCCCGCCTGGCATTATGCCCAGTACATGACCTTATGGGACTTTCCTACTTGGCAGTACATCTACGTATTAGTCATCGCTATTACCATGGTGATGCGGTTTTGGCAGTACATCAATGGGCGTGGATAGCGGTTTGACTCACGGGGATTTCCAAGTCTCCACCCCATTGACGTCAATGGGAGTTTGTTTTGGCACCAAAATCAACGGGACTTTCCAAAATGTCGTAACAACTCCGCCCCATTGACGCAAATGGGCGGTAGGCGTGTACGGTGGGAGGTCTATATAAGCAGAGCTCGTTTAGTGAACCGTCAGATCGCCTGGAGACGCCATCCACGCTGTTTTGACCTCCATAGAAGACACCGGGACCGATCCAGCCTCCCCTCGAAGCTGATCCTGAGAACTTCAGGGTGAGTCTATGGGACCCTTGATGTTTTCTTTCCCCTTCTTTTCTATGGTTAAGTTCATGTCATAGGAAGGGGAGAAGTAACAGGGTACACATATTGACCAAATCAGGGTAATTTTGCATTTGTAATTTTAAAAAATGCTTTCTTCTTTTAATATACTTTTTTGTTTATCTTATTTCTAATACTTTCCCTAATCTCTTTCTTTCAGGGCAATAATGATACAATGTATCATGCCTCTTTGCACCATTCTAAAGAATAACAGTGATAATTTCTGGGTTAAGGCAATAGCAATATTTCTGCATATAAATATTTCTGCATATAAATTGTAACTGATGTAAGAGGTTTCATATTGCTAATAGCAGCTACAATCCAGCTACCATTCTGCTTTTATTTTATGGTTGGGATAAGGCTGGATTATTCTGAGTCCAAGCTAGGCCCTTTTGCTAATCATGTTCATACCTCTTATCTTCCTCCCACAGCTCCTGGGCAACGTGCTGGTCTGTGTGCTGGCCCATCACTTTGGCAAAGAATTCACTAGTGATTTCGCCGCCACCATGGTGAGCAAGGGCGAGGAGCTGTTCACCGGGGTGGTGCCCATCCTGGTCGAGCTGGACGGCGACGTAAACGGCCACAAGTTCAGCGTGTCCGGCGAGGGCGAGGGCGATGCCACCTACGGCAAGCTGACCCTGAAGTTCATCTGCACCACCGGCAAGCTGCCCGTGCCCTGGCCCACCCTCGTGACCACCCTGACCTACGGCGTGCAGTGCTTCAGCCGCTACCCCGACCACATGAAGCAGCACGACTTCTTCAAGTCCGCCATGCCCGAAGGCTACGTCCAGGAGCGCACCATCTTCTTCAAGGACGACGGCAACTACAAGACCCGCGCCGAGGTGAAGTTCGAGGGCGACACCCTGGTGAACCGCATCGAGCTGAAGGGCATCGACTTCAAGGAGGACGGCAACATCCTGGGGCACAAGCTGGAGTACAACTACAACAGCCACAACGTCTATATCATGGCCGACAAGCAGAAGAACGGCATCAAGGTGAACTTCAAGATCCGCCACAACATCGAGGACGGCAGCGTGCAGCTCGCCGACCACTACCAGCAGAACACCCCCATCGGCGACGGCCCCGTGCTGCTGCCCGACAACCACTACCTGAGCACCCAGTCCGCCCTGAGCAAAGACCCCAACGAGAAGCGCGATCACATGGTCCTGCTGGAGTTCGTGACCGCCGCCGGGATCACTCTCGGCATGGACGAGCTGTACAAGTAAGATATCTTATAAGTNNNNNNACAAGCTTATCGATAATCAACCTCTGGATTACAAAATTTGTGAAAGATTGACTGGTATTCTTAACTATGTTGCTCCTTTTACGCTATGTGGATACGCTGCTTTAATGCCTTTGTATCATGCTATTGCTTCCCGTATGGCTTTCATTTTCTCCTCCTTGTATAAATCCTGGTTGCTGTCTCTTTATGAGGAGTTGTGGCCCGTTGTCAGGCAACGTGGCGTGGTGTGCACTGTGTTTGCTGACGCAACCCCCACTGGTTGGGGCATTGCCACCACCTGTCAGCTCCTTTCCGGGACTTTCGCTTTCCCCCTCCCTATTGCCACGGCGGAACTCATCGCCGCCTGCCTTGCCCGCTGCTGGACAGGGGCTCGGCTGTTGGGCACTGACAATTCCGTGGTGTTGTCGGGGAAATCATCGTCCTTTCCTTGGCTGCTCGCCTGTGTTGCCACCTGGATTCTGCGCGGGACGTCCTTCTGCTACGTCCCTTCGGCCCTCAATCCAGCGGACCTTCCTTCCCGCGGCCTGCTGCCGGCTCTGCGGCCTCTTCCGCGTCTTCGCCTTCGCCCTCAGACGAGTCGGATCTCCCTTTGGGCCGCCTCCCCGCATCGATACCGTCGACTCGCTGATCAGCCTCGACTGTGCCTTCTAGTTGCCAGCCATCTGTTGTTTGCCCCTCCCCCGTGCCTTCCTTGACCCTGGAAGGTGCCACTCCCACTGTCCTTTCCTAATAAAATGAGGAAATTGCATCGCATTGTCTGAGTAGGTGTCATTCTATTCTGGGGGGTGGGGTGGGGCAGGACAGCAAGGGGGAGGATTGGGAAGACAATAGCAGGCATGCTGGGGATGCGGTGGGCTCTATGGCTTCTGAGGCGGAAAGAACCAGCTGGGGCTCGACTAGAGGAACCCCTAGTGATGGAGTTGGCCACTCCCTCTCTGCGCGCTCGCTCGCTCACTGAGGCCGGGCGACCAAAGGTCGCCCGACGCCCGGGCTTTGCCCGGGCGGCCTCAGTGAGCGAGCGAGCGCGCAGAGAGGGAGTGGCCAA |
| --- | --- |
| ssAAV-ITR-CMV-mCherry-WPRE-BC-BGHpA-ITR | TTGGCCACTCCCTCTCTGCGCGCTCGCTCGCTCACTGAGGCCGGGCGACCAAAGGTCGCCCGACGCCCGGGCTTTGCCCGGGCGGCCTCAGTGAGCGAGCGAGCGCGCAGAGAGGGAGTGGCCAACTCCATCACTAGGGGTTCCTGGTACCGAGGACATTGATTATTGACTAGTTATTAATAGTAATCAATTACGGGGTCATTAGTTCATAGCCCATATATGGAGTTCCGCGTTACATAACTTACGGTAAATGGCCCGCCTGGCTGACCGCCCAACGACCCCCGCCCATTGACGTCAATAATGACGTATGTTCCCATAGTAACGCCAATAGGGACTTTCCATTGACGTCAATGGGTGGAGTATTTACGGTAAACTGCCCACTTGGCAGTACATCAAGTGTATCATATGCCAAGTACGCCCCCTATTGACGTCAATGACGGTAAATGGCCCGCCTGGCATTATGCCCAGTACATGACCTTATGGGACTTTCCTACTTGGCAGTACATCTACGTATTAGTCATCGCTATTACCATGGTGATGCGGTTTTGGCAGTACATCAATGGGCGTGGATAGCGGTTTGACTCACGGGGATTTCCAAGTCTCCACCCCATTGACGTCAATGGGAGTTTGTTTTGGCACCAAAATCAACGGGACTTTCCAAAATGTCGTAACAACTCCGCCCCATTGACGCAAATGGGCGGTAGGCGTGTACGGTGGGAGGTCTATATAAGCAGAGCTCGTTTAGTGAACCGTCAGATCGCCTGGAGACGCCATCCACGCTGTTTTGACCTCCATAGAAGACACCGGGACCGATCCAGCCTCCCCTCGAAGCTGATCCTGAGAACTTCAGGGTGAGTCTATGGGACCCTTGATGTTTTCTTTCCCCTTCTTTTCTATGGTTAAGTTCATGTCATAGGAAGGGGAGAAGTAACAGGGTACACATATTGACCAAATCAGGGTAATTTTGCATTTGTAATTTTAAAAAATGCTTTCTTCTTTTAATATACTTTTTTGTTTATCTTATTTCTAATACTTTCCCTAATCTCTTTCTTTCAGGGCAATAATGATACAATGTATCATGCCTCTTTGCACCATTCTAAAGAATAACAGTGATAATTTCTGGGTTAAGGCAATAGCAATATTTCTGCATATAAATATTTCTGCATATAAATTGTAACTGATGTAAGAGGTTTCATATTGCTAATAGCAGCTACAATCCAGCTACCATTCTGCTTTTATTTTATGGTTGGGATAAGGCTGGATTATTCTGAGTCCAAGCTAGGCCCTTTTGCTAATCATGTTCATACCTCTTATCTTCCTCCCACAGCTCCTGGGCAACGTGCTGGTCTGTGTGCTGGCCCATCACTTTGGCAAAGAATTCACTAGTGATTTCGCCGCCACCATGGTGAGCAAGGGCGAGGAGGATAACATGGCCATCATCAAGGAGTTCATGCGCTTCAAGGTGCACATGGAGGGCTCCGTGAACGGCCACGAGTTCGAGATCGAGGGCGAGGGCGAGGGCCGCCCCTACGAGGGCACCCAGACCGCCAAGCTGAAGGTGACCAAGGGTGGCCCCCTGCCCTTCGCCTGGGACATCCTGTCCCCTCAGTTCATGTACGGCTCCAAGGCCTACGTGAAGCACCCCGCCGACATCCCCGACTACTTGAAGCTGTCCTTCCCCGAGGGCTTCAAGTGGGAGCGCGTGATGAACTTCGAGGACGGCGGCGTGGTGACCGTGACCCAGGACTCCTCCCTGCAGGACGGCGAGTTCATCTACAAGGTGAAGCTGCGCGGCACCAACTTCCCCTCCGACGGCCCCGTAATGCAGAAGAAGACCATGGGCTGGGAGGCCTCCTCCGAGCGGATGTACCCCGAGGACGGCGCCCTGAAGGGCGAGATCAAGCAGAGGCTGAAGCTGAAGGACGGCGGCCACTACGACGCTGAGGTCAAGACCACCTACAAGGCCAAGAAGCCCGTGCAGCTGCCCGGCGCCTACAACGTCAACATCAAGTTGGACATCACCTCCCACAACGAGGACTACACCATCGTGGAACAGTACGAACGCGCCGAGGGCCGCCACTCCACCGGCGGCATGGACGAGCTGTACAAGTAGAAGCTTATCGATAATCAACCTCTGGATTACAAAATTTGTGAAAGATTGACTGGTATTCTTAACTATGTTGCTCCTTTTACGCTATGTGGATACGCTGCTTTAATGCCTTTGTATCATGCTATTGCTTCCCGTATGGCTTTCATTTTCTCCTCCTTGTATAAATCCTGGTTGCTGTCTCTTTATGAGGAGTTGTGGCCCGTTGTCAGGCAACGTGGCGTGGTGTGCACTGTGTTTGCTGACGCAACCCCCACTGGTTGGGGCATTGCCACCACCTGTCAGCTCCTTTCCGGGACTTTCGCTTTCCCCCTCCCTATTGCCACGGCGGAACTCATCGCCGCCTGCCTTGCCCGCTGCTGGACAGGGGCTCGGCTGTTGGGCACTGACAATTCCGTGGTGTTGTCGGGGAAATCATCGTCCTTTCCTTGGCTGCTCGCCTGTGTTGCCACCTGGATTCTGCGCGGGACGTCCTTCTGCTACGTCCCTTCGGCCCTCAATCCAGCGGACCTTCCTTCCCGCGGCCTGCTGCCGGCTCTGCGGCCTCTTCCGCGTCTTCGCCTTCGCCCTCAGACGAGTCGGATCTCCCTTTGGGCCGCCTCCCCGCATCGATANNNNNNNNNNNNNNNNNNNNNNNCCGTCGACTCGCTGATCAGCCTCGACTGTGCCTTCTAGTTGCCAGCCATCTGTTGTTTGCCCCTCCCCCGTGCCTTCCTTGACCCTGGAAGGTGCCACTCCCACTGTCCTTTCCTAATAAAATGAGGAAATTGCATCGCATTGTCTGAGTAGGTGTCATTCTATTCTGGGGGGTGGGGTGGGGCAGGACAGCAAGGGGGAGGATTGGGAAGACAATAGCAGGCATGCTGGGGATGCGGTGGGCTCTATGGCTTCTGAGGCGGAAAGAACCAGCTGGGGCTCGACTAGAGGAACCCCTAGTGATGGAGTTGGCCACTCCCTCTCTGCGCGCTCGCTCGCTCACTGAGGCCGGGCGACCAAAGGTCGCCCGACGCCCGGGCTTTGCCCGGGCGGCCTCAGTGAGCGAGCGAGCGCGCAGAGAGGGAGTGGCCAA |
| scAAV-ITR-hSYNrv-p40- eGFP—SV40pA-ITR delta trs | GGCCACTCCCTCTCTGCGCGCTCGCTCGCTCACTGAGGCCGGGCGACCAAAGGTCGCCCGACGCCCGGGCTTTGCCCGGGCGGCCTCAGTGAGCGAGCGAGCGCGCAGAGAGGGAGTGGCCAACTCCATCACTAGGGGTTCCTGGAGGGGTGGAGTCGTGACCTAGGGTACCTTCTCGACTGCGCTCTCAGGCACGACACGACTCCTCCGCTGCCCACCGCAGACTGAGGCAGCGCTGAGTCGCCGGCGCCGCAGCGCAGATGGTCGCGCCCGTGCCCCCCTATCTCGCGCCTCGCGTGGTGCGGTCCGGCTGGGCCGGCGGCGGCGCGGACGCGACCAAGGTGGCCGGGAAGGGGAGTTTGCGGGGGACCGGCGAGTGACGTCAGCGCGCCTTCAGTGCTGAGGCGGCGGTGGCGCGCGCCGCCAGGCGGGGGCGAAGGCACTGTCCGCGGTGCTGAAGCTGGCAGTGCGCACGCGCCTCGCCGCATCCTGTTTCCCCTCCCCCTCTCTGATAGGGGATGCGCAATTTGGGGAATGGGGGTTGGGTGCTTGTCCAGTGGGTCGGGGTCGGTCGTCAGGTAGGCACCCCCACCCCGCCTCATCCTGGTCCTAAAACCCACTTGCACTCATACGCAGGGCCCTCTGCAGTCTAGACACACGCGTAGTCCCTCGCATCGCCTGCGCTCCCTCCGCAGTTTCAATTGTAAGGTCACCAAGCAGGAAGTCAAAGACTTTTTCCGGTGGGCAAAGGATCACGTGGTTGAGGTGGAGCATGAATTCTACGTCAAAAAGGGTGGAGCCAAGAAAAGACCCGCCCCCAGTGACGCAGATATAAGTGAGCCCAAACGGGTGCGCGAGTCGGATCCACCGGTCGCCACCATGGTGAGCAAGGGCGAGGAGCTGTTCACCGGGGTGGTGCCCATCCTGGTCGAGCTGGACGGCGACGTAAACGGCCACAAGTTCAGCGTGTCCGGCGAGGGCGAGGGCGATGCCACCTACGGCAAGCTGACCCTGAAGTTCATCTGCACCACCGGCAAGCTGCCCGTGCCCTGGCCCACCCTCGTGACCACCCTGACCTACGGCGTGCAGTGCTTCAGCCGCTACCCCGACCACATGAAGCAGCACGACTTCTTCAAGTCCGCCATGCCCGAAGGCTACGTCCAGGAGCGCACCATCTTCTTCAAGGACGACGGCAACTACAAGACCCGCGCCGAGGTGAAGTTCGAGGGCGACACCCTGGTGAACCGCATCGAGCTGAAGGGCATCGACTTCAAGGAGGACGGCAACATCCTGGGGCACAAGCTGGAGTACAACTACAACAGCCACAACGTCTATATCATGGCCGACAAGCAGAAGAACGGCATCAAGGTGAACTTCAAGATCCGCCACAACATCGAGGACGGCAGCGTGCAGCTCGCCGACCACTACCAGCAGAACACCCCCATCGGCGACGGCCCCGTGCTGCTGCCCGACAACCACTACCTGAGCACCCAGTCCGCCCTGAGCAAAGACCCCAACGAGAAGCGCGATCACATGGTCCTGCTGGAGTTCGTGACCGCCGCCGGGATCACTCTCGGCATGGACGAGCTGTACAAGTAAAGCGGCCTTCAGATCCTCACCTGCGATCTCGATGCTTTATTTGTGAAATTTGTGATGCTATTGCTTTATTTGTAACCATTATAAGCTGCAATAAACAAGTTAACAACAACAATTGCATTCATTTTATGTTTCAGGTTCAGGGGGAGGTGTGGGAGGTTTTTTAAACTAGTCCACTCCCTCTCTGCGCGCTCGCTCGCTCACTGAGGCCGGGCGACCAAAGGTCGCCCGACGCCCGGGCTTTGCCCGGGCGGCCTCAGTGAGCGAGCGAGCGCGCAGAGAGGGA |

# Supplementary Table 5. Barcode counts of triple barcoded Retina AAV Kit 2 input mix

| **Barcode** | **read counts** | **percentage of all reads** | **Normalization co-efficient for each barcode** |
| --- | --- | --- | --- |
| 01_AAV2_A - AAAACATAAATTGCATTGTTTTC | 10098 | 0.85% | 2.29 |
| 01_AAV2_B - TTTTTCCCCTTTCTCCCAACCGT | 9246 | 0.78% | 2.51 |
| 01_AAV2_C - TGACCTCTCGACAATCTCGATCA | 8362 | 0.71% | 2.77 |
| 02_AAV5_A - ATCGCGATACTTGTACTTCTAGC | 15668 | 1.32% | 1.48 |
| 02_AAV5_B - TAAAAGTAAGCAATTCTAAAAGC | 12658 | 1.07% | 1.83 |
| 02_AAV5_C - TTTGTCTTCATGTCTTTTTCTTT | 14846 | 1.26% | 1.57 |
| 03_AAV8_A - AACACTGAAAAAGAATCAATGTA | 44001 | 3.72% | 0.53 |
| 03_AAV8_B - AGGTAAGGCGAAAATGGGGCGGG | 28184 | 2.38% | 0.82 |
| 03_AAV8_C - ACAAACAGGAAAGAAAATCGCGG | 29955 | 2.53% | 0.77 |
| 04_Anc80_A - CCAGCGTGTAGAAAGGCTAGGGT | 8220 | 0.70% | 2.81 |
| 04_Anc80_B - CGATCCAGACATCCCGAACGCAG | 7962 | 0.68% | 2.90 |
| 04_Anc80_C - GGGTGACAAGTACCTGTTCAGGT | 7566 | 0.64% | 3.06 |
| 05_shH10_A - CCATGACTTACGTGTGGATCAGG | 21674 | 1.83% | 1.07 |
| 05_shH10_B - CAGTTTAAGGGGGACGGGTAAAA | 19569 | 1.66% | 1.18 |
| 05_shH10_C - CACTGAGAGAAGGTCAAACCAAA | 28776 | 2.44% | 0.81 |
| 06_7m8_A - AAGCCTGTAAAGCGCTGGAAAGG | 22723 | 1.93% | 1.02 |
| 06_7m8_B - GTGAGATGGTGCGCTGGGCCATG | 22606 | 1.92% | 1.02 |
| 06_7m8_C - GGAGGAGCGGGCAGAGAGGGAAG | 36313 | 3.08% | 0.64 |
| 07_1.1_A - AAAAAGAAACAGTCGAATAACAA | 30216 | 2.56% | 0.77 |
| 07_1.1_B - GGGTGTTTCCCGGGTGTTGTGCG | 27603 | 2.34% | 0.84 |
| 07_1.1_C - CATGCTCCGCCCTCCCCTCTCCC | 22798 | 1.93% | 1.02 |
| 08_1.2_A - CCATCCAGACCAACTTATCCCCC | 32117 | 2.72% | 0.72 |
| 08_1.2_B - AATGAGCACGGAAATGTAATAAG | 29388 | 2.49% | 0.79 |
| 08_1.2_C - CCGAGAAACGCGGGACTGGAGGT | 46203 | 3.91% | 0.50 |
| 09_1.3_A - AATGGGAGAGGTGCGACATGGCG | 20140 | 1.71% | 1.15 |
| 09_1.3_B - GGTGGTGGCAGCTTTGGGGTTGA | 21540 | 1.82% | 1.08 |
| 09_1.3_C - GTAGATGACGAGACCACGGTAAC | 17894 | 1.52% | 1.29 |
| 10_L1_A - TTACAAAAGACAATGTAAAGCCC | 19446 | 1.65% | 1.19 |
| 10_L1_B - TCGATATGCTGCGGATGGCGAAA | 24976 | 2.12% | 0.93 |
| 10_L1_C - AACTAAACACACAACCCACCTAG | 20563 | 1.75% | 1.12 |
| 11_L2_A - TTGTTGTTGATTTTCTTTATTTA | 22118 | 1.87% | 1.05 |
| 11_L2_B - CTAAACGGCAGTAAAATGAGTAA | 26782 | 2.27% | 0.86 |
| 11_L2_C - TACTTTGAAGCAGACAGTCCGGC | 18024 | 1.53% | 1.28 |
| 12_L3_A - AGGCAACCGGTTGATAGGCAGAG | 28241 | 2.39% | 0.82 |
| 12_L3_B - CCGAATCGAGTAAACTGGAACAG | 29067 | 2.46% | 0.80 |
| 12_L3_C - AACTAGAATTGATAAACCAACCA | 24761 | 2.10% | 0.93 |
| 13_L4_A - GCGAAAAAGCAGTGAGGGACGAC | 32155 | 2.72% | 0.72 |
| 13_L4_B - TTTTATCCATTATCATTACCATT | 24296 | 2.06% | 0.95 |
| 13_L4_C - GCACAACAAACTATGGCGCCACA | 27285 | 2.31% | 0.85 |
| 14_L5_A - CCGAGAGGAGTTAACACAGAGAG | 23647 | 2.00% | 0.98 |
| 14_L5_B - ATAGCATGTGAGTCGGGGTAATT | 32522 | 2.76% | 0.71 |
| 14_L5_C - GCGCACCAGGGCAGCGAAGAGTA | 28848 | 2.45% | 0.80 |
| 15_M1_A - AGATCTGCACGCAACAGAGCCGA | 20989 | 1.78% | 1.10 |
| 15_M1_B - ACGAGGTTACGGGGGAGCTTAAT | 20764 | 1.76% | 1.11 |
| 15_M1_C - GCACAGAGGTCCGAGTCGCATAC | 22791 | 1.93% | 1.01 |
| 16_M2_A - GGCCGTTGAAGGTGGAAGTAGAT | 26268 | 2.22% | 0.88 |
| 16_M2_B - TAAAACCAGCGATCCCGACCAGT | 25144 | 2.13% | 0.92 |
| 16_M2_C - AGTTTCGAGAAACCACGAGCATG | 19985 | 1.69% | 1.16 |
| 18_M4_A - CCCCCCTGTAATTTCTTGCACGG | 19292 | 1.64% | 1.20 |
| 18_M4_B - AAGTCGCAAAAACTGAAAAAGTA | 18003 | 1.53% | 1.28 |
| 18_M4_C - AAACCCTACTGAGAAACCGCGCA | 28135 | 2.39% | 0.82 |

# Supplementary Table 6. Barcode counts of triple barcoded Retina AAV Kit 2 DNA sample for one murine retina

| **Barcode** | **read counts** | **normalized counts** | **percentage** |
| --- | --- | --- | --- |
| 01_AAV2_A - AAAACATAAATTGCATTGTTTTC | 88850 | 203867.37 | 21.61% |
| 01_AAV2_B - TTTTTCCCCTTTCTCCCAACCGT | 89007 | 223028.40 | 23.64% |
| 01_AAV2_C - TGACCTCTCGACAATCTCGATCA | 75302 | 208615.41 | 22.11% |
| 02_AAV5_A - ATCGCGATACTTGTACTTCTAGC | 10137 | 15032.39 | 1.59% |
| 02_AAV5_B - TAAAAGTAAGCAATTCTAAAAGC | 7922 | 14509.15 | 1.54% |
| 02_AAV5_C - TTTGTCTTCATGTCTTTTTCTTT | 11210 | 17548.83 | 1.86% |
| 03_AAV8_A - AACACTGAAAAAGAATCAATGTA | 10505 | 5534.14 | 0.59% |
| 03_AAV8_B - AGGTAAGGCGAAAATGGGGCGGG | 7195 | 5927.61 | 0.63% |
| 03_AAV8_C - ACAAACAGGAAAGAAAATCGCGG | 6865 | 5313.54 | 0.56% |
| 04_Anc80_A - CCAGCGTGTAGAAAGGCTAGGGT | 230 | 646.89 | 0.07% |
| 04_Anc80_B - CGATCCAGACATCCCGAACGCAG | 229 | 665.02 | 0.07% |
| 04_Anc80_C - GGGTGACAAGTACCTGTTCAGGT | 295 | 902.20 | 0.10% |
| 05_shH10_A - CCATGACTTACGTGTGGATCAGG | 15016 | 16103.88 | 1.71% |
| 05_shH10_B - CAGTTTAAGGGGGACGGGTAAAA | 2658 | 3140.13 | 0.33% |
| 05_shH10_C - CACTGAGAGAAGGTCAAACCAAA | 18800 | 15145.52 | 1.61% |
| 06_7m8_A - AAGCCTGTAAAGCGCTGGAAAGG | 51544 | 52481.66 | 5.56% |
| 06_7m8_B - GTGAGATGGTGCGCTGGGCCATG | 51424 | 52632.53 | 5.58% |
| 06_7m8_C - GGAGGAGCGGGCAGAGAGGGAAG | 82289 | 52438.94 | 5.56% |
| 07_1.1_A - AAAAAGAAACAGTCGAATAACAA | 1339 | 1025.32 | 0.11% |
| 07_1.1_B - GGGTGTTTCCCGGGTGTTGTGCG | 1209 | 1014.47 | 0.11% |
| 07_1.1_C - CATGCTCCGCCCTCCCCTCTCCC | 1081 | 1099.55 | 0.12% |
| 08_1.2_A - CCATCCAGACCAACTTATCCCCC | 833 | 601.19 | 0.06% |
| 08_1.2_B - AATGAGCACGGAAATGTAATAAG | 587 | 462.39 | 0.05% |
| 08_1.2_C - CCGAGAAACGCGGGACTGGAGGT | 1045 | 524.04 | 0.06% |
| 09_1.3_A - AATGGGAGAGGTGCGACATGGCG | 2799 | 3213.20 | 0.34% |
| 09_1.3_B - GGTGGTGGCAGCTTTGGGGTTGA | 16310 | 17596.23 | 1.86% |
| 09_1.3_C - GTAGATGACGAGACCACGGTAAC | 2279 | 2944.15 | 0.31% |
| 10_L1_A - TTACAAAAGACAATGTAAAGCCC | 3705 | 4403.38 | 0.47% |
| 10_L1_B - TCGATATGCTGCGGATGGCGAAA | 4907 | 4546.82 | 0.48% |
| 10_L1_C - AACTAAACACACAACCCACCTAG | 3840 | 4314.71 | 0.46% |
| 11_L2_A - TTGTTGTTGATTTTCTTTATTTA | 45 | 47.07 | 0.00% |
| 11_L2_B - CTAAACGGCAGTAAAATGAGTAA | 42 | 36.27 | 0.00% |
| 11_L2_C - TACTTTGAAGCAGACAGTCCGGC | 28 | 35.93 | 0.00% |
| 12_L3_A - AGGCAACCGGTTGATAGGCAGAG | 93 | 76.24 | 0.01% |
| 12_L3_B - CCGAATCGAGTAAACTGGAACAG | 72 | 57.31 | 0.01% |
| 12_L3_C - AACTAGAATTGATAAACCAACCA | 89 | 83.12 | 0.01% |
| 13_L4_A - GCGAAAAAGCAGTGAGGGACGAC | 706 | 508.36 | 0.05% |
| 13_L4_B - TTTTATCCATTATCATTACCATT | 644 | 613.31 | 0.06% |
| 13_L4_C - GCACAACAAACTATGGCGCCACA | 448 | 379.90 | 0.04% |
| 14_L5_A - CCGAGAGGAGTTAACACAGAGAG | 539 | 527.75 | 0.06% |
| 14_L5_B - ATAGCATGTGAGTCGGGGTAATT | 660 | 469.75 | 0.05% |
| 14_L5_C - GCGCACCAGGGCAGCGAAGAGTA | 549 | 440.31 | 0.05% |
| 15_M1_A - AGATCTGCACGCAACAGAGCCGA | 1287 | 1417.46 | 0.15% |
| 15_M1_B - ACGAGGTTACGGGGGAGCTTAAT | 1225 | 1364.49 | 0.14% |
| 15_M1_C - GCACAGAGGTCCGAGTCGCATAC | 1960 | 1989.28 | 0.21% |
| 16_M2_A - GGCCGTTGAAGGTGGAAGTAGAT | 74 | 65.23 | 0.01% |
| 16_M2_B - TAAAACCAGCGATCCCGACCAGT | 69 | 63.47 | 0.01% |
| 16_M2_C - AGTTTCGAGAAACCACGAGCATG | 52 | 60.24 | 0.01% |
| 18_M4_A - CCCCCCTGTAATTTCTTGCACGG | 34 | 40.77 | 0.00% |
| 18_M4_B - AAGTCGCAAAAACTGAAAAAGTA | 27 | 34.66 | 0.00% |
| 18_M4_C - AAACCCTACTGAGAAACCGCGCA | 25 | 20.54 | 0.00% |


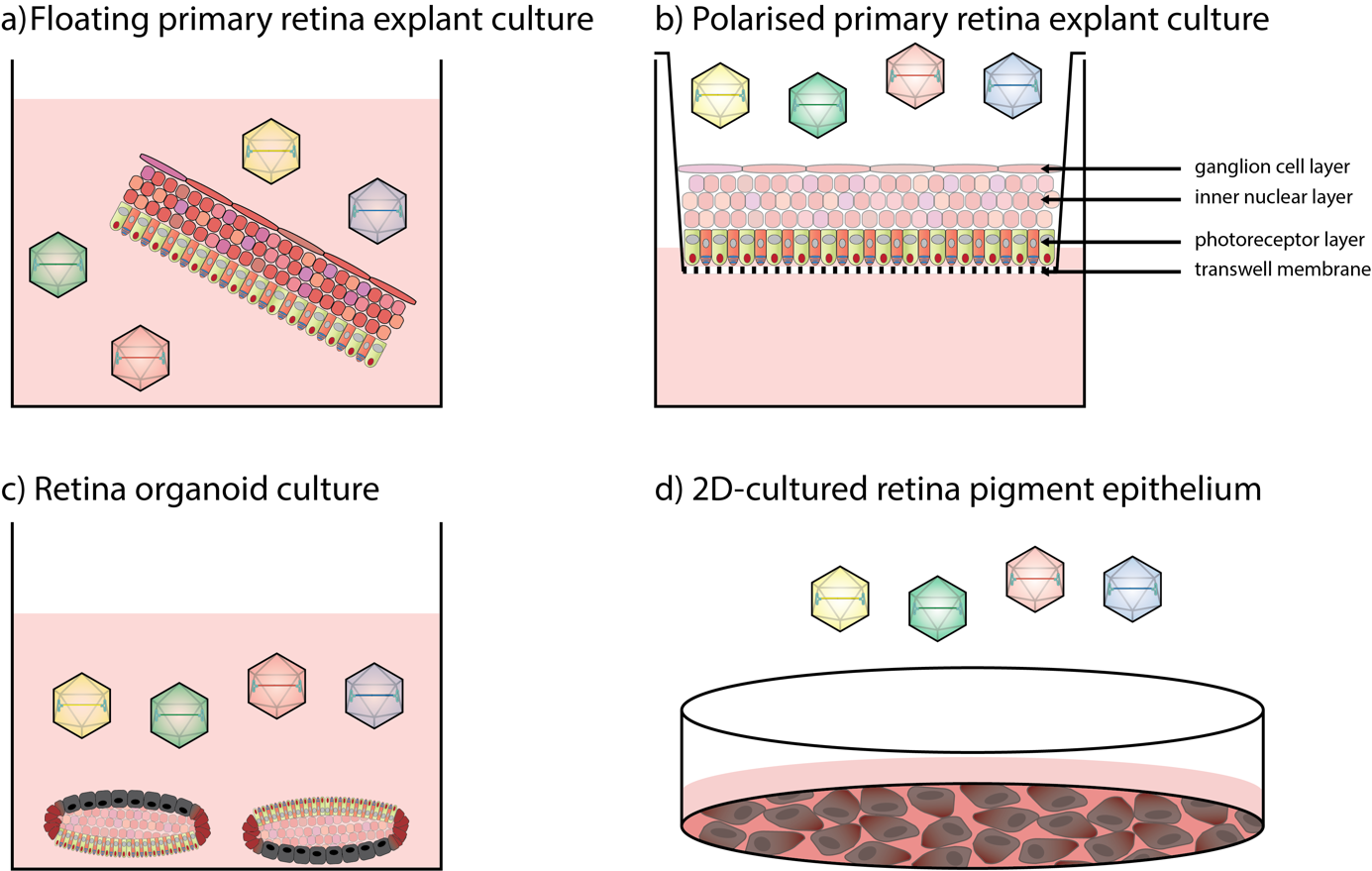


# Supplementary Figure 1. Overview of retina models used in this study. a) Primary human retina holepunches submerged in culture media and referred to as “floating” explants in the presented study. Vectors were exposed to the explant from all sides. b) Polarised primary retina explants cultured on transwell inserts. The photoreceptor layer is in contact with culture media through the transwell membrane. Vectors contacted the tissue only from the ganglion cell layer. c) Submerged human induced pluripotent stem cell (hiPSC)-derived retina organoid cultures. AAVs could access organoids from all sides. d) 2D-cultured retina pigment epithelium derived from primary human tissue or differentiated from hiPSC.


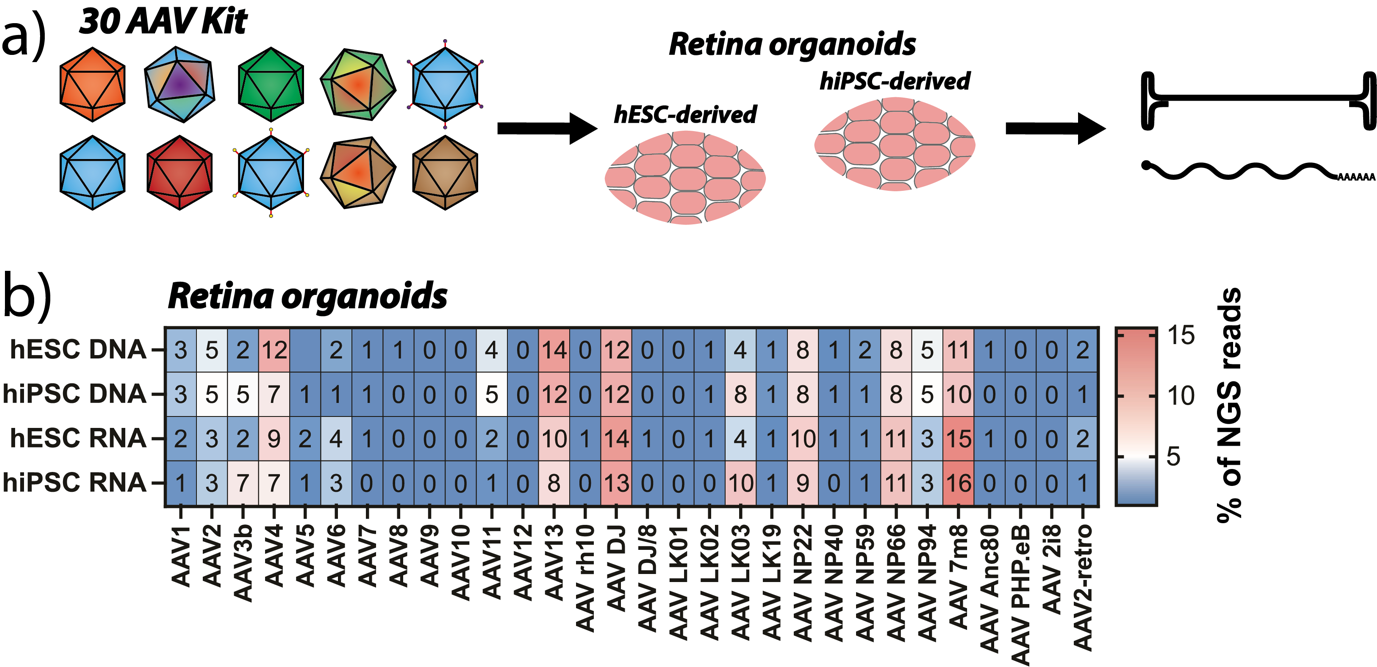


# Supplementary Figure 2. Results from the 30 ‘AAV Kit’ on retina organoids

**(a**) Schematic representation of the performed experiment. (**b**) Results of NGS-based quantification of AAV-delivered DNA and RNA/cDNA in retina organoids. Values are given as percentage of total reads. Number of replicates for each model: n=1.


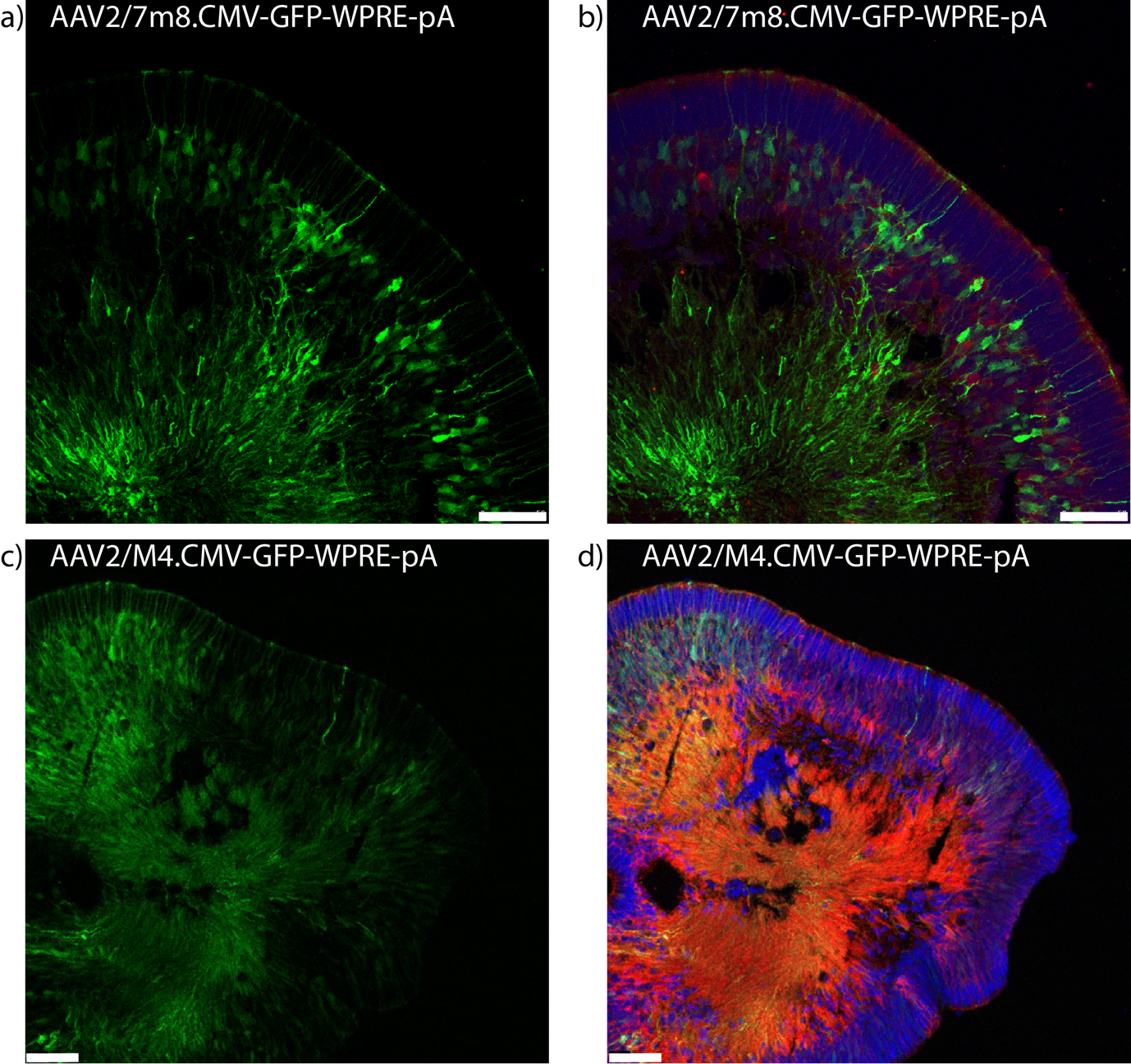


# Supplementary Figure 3. Detailed analysis of the transduction of AAV2-M4 in retinal organoids. Transduction of iPSC-derived retinal organoids with 5×10^10^ vector genomes using AAV-7m8 and AAV2-M4; n=1. (a-b) Representative confocal images showing AAV2-7m8 transduction with native CMV-driven eGFP expression [a], DAPI, and an overlay of eGFP (green), DAPI (blue) and cone arrestin (red) [b]. (c-d) Representative confocal images showing AAV2-M4 transduction with native CMV-driven eGFP expression [c], and an overlay of eGFP (green), DAPI (blue), and cellular retinaldehyde-binding protein (red) [d].


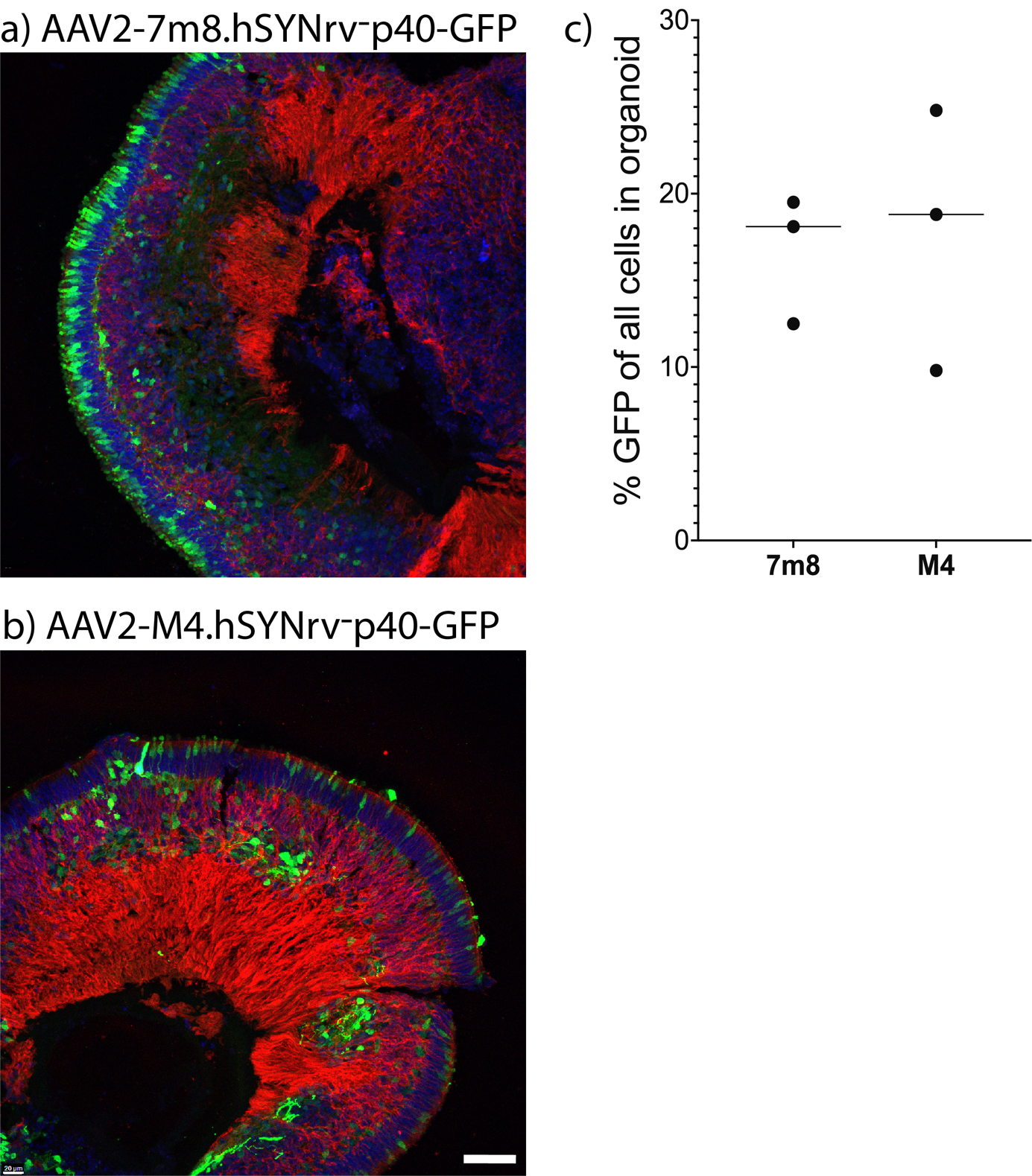


# Supplementary Figure 4. Comparing the transduction of AAV-7m8 and AAV2-M4 using the hSYNrv-p40 promoter. (a) Representative microscopy image showing transduction of AAV-7m8 capsid delivering hSYNrv-p40-driven eGFP green: anti-GFP antibody-counterstained eGFP expression; red: cellular retinaldehyde-binding protein; blue: DAPI; n=1. (b) Flow cytometry showing eGFP expression of all DAPI-negative cells in three independently prepared retinal organoid cultures; n=3.


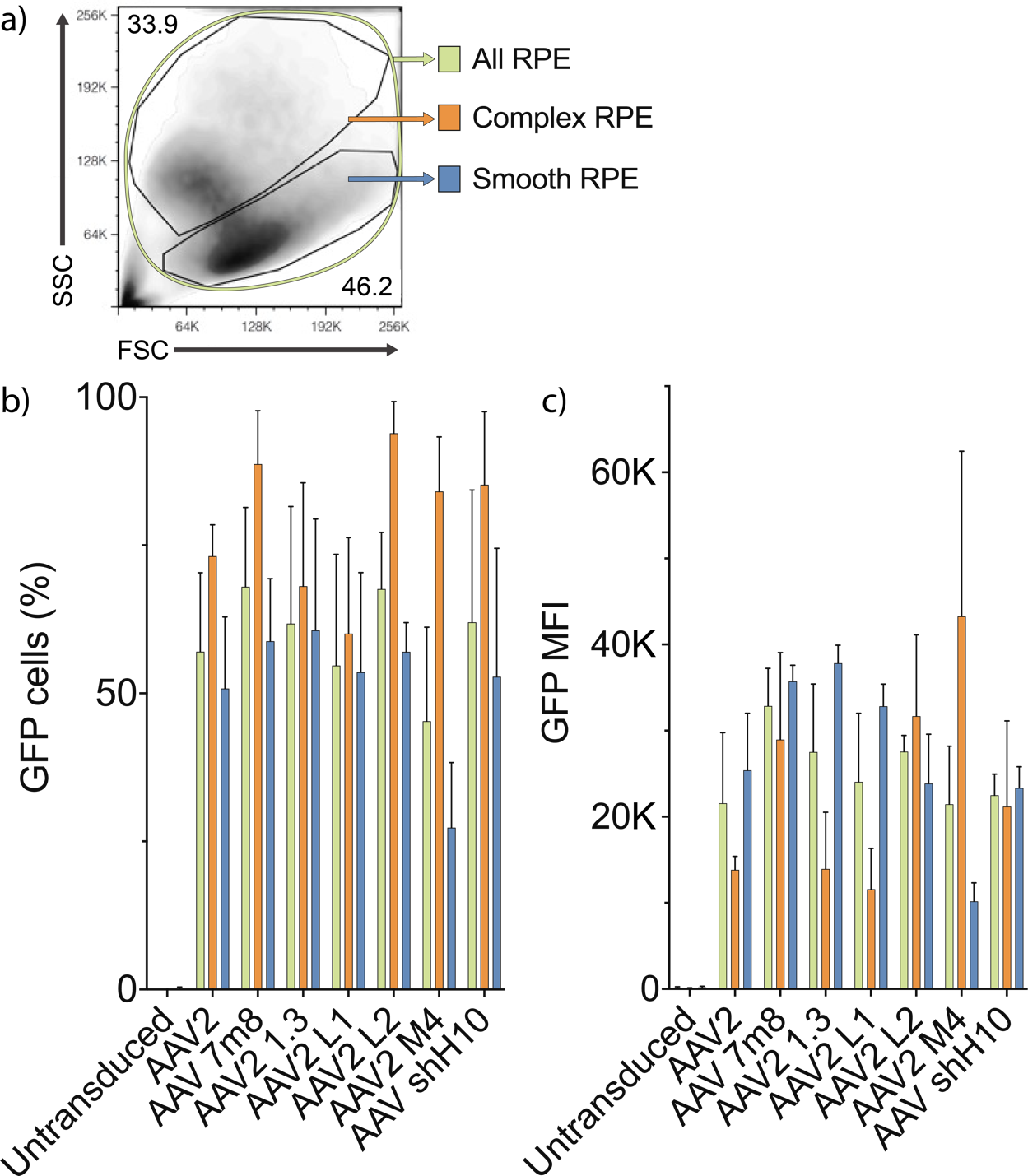


# Supplementary Figure 5. Detailed analysis of the transduction of the top candidates in iPSC-derived retinal pigment epithelium. (a) Representative graph of cell populations within all DAPI-negative cells of iPSC-derived RPE. (b-c) eGFP positive cells [b] and mean fluorescent intensity [c] within the three cell populations defined in [a] shown for the indicated novel and benchmark AAV capsids; n=2. Green: all DAPI-negative cells; orange: granular (high SSC) cells; blue: smooth (low SSC) cells.


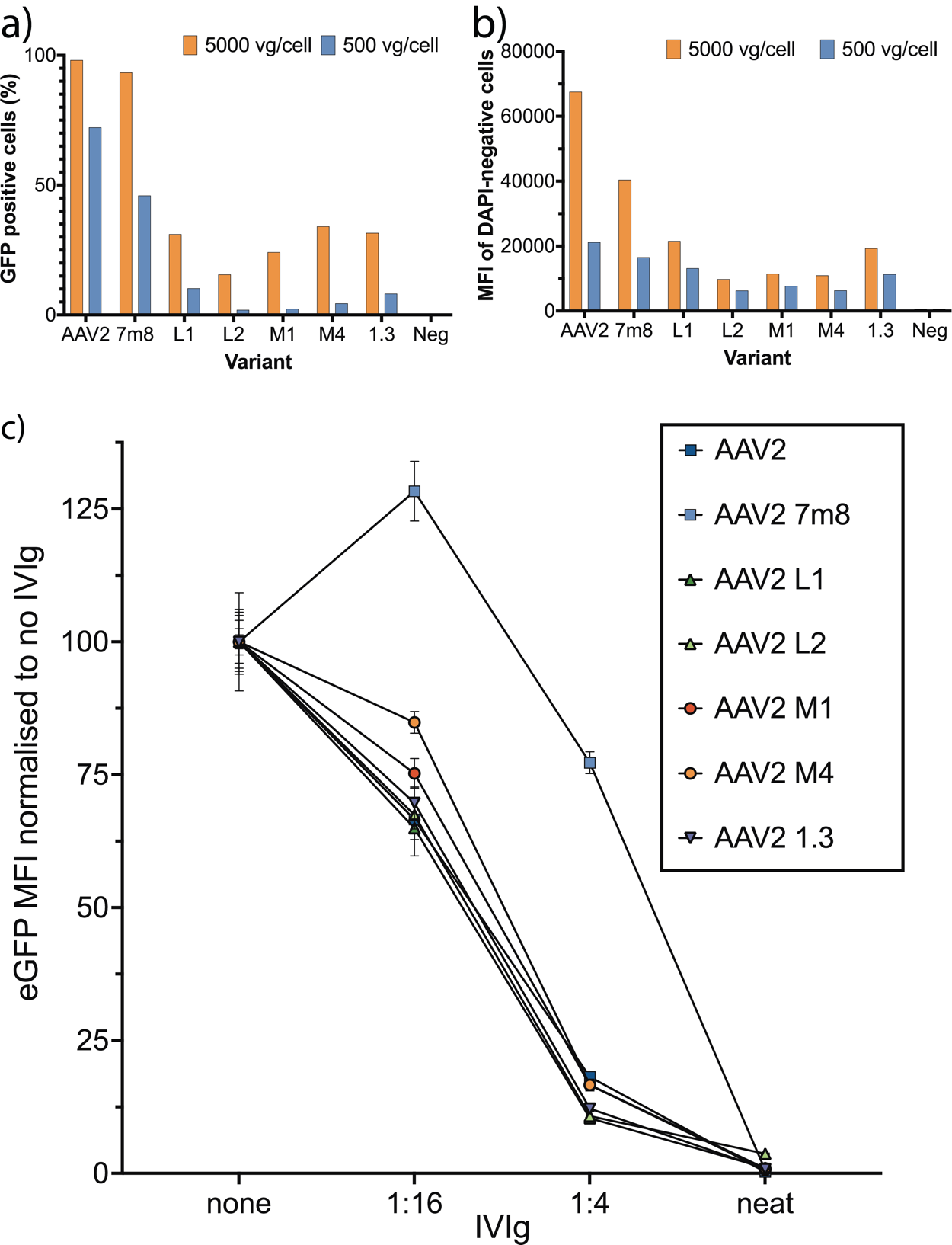


# Supplementary Figure 6. Resistance to neutralisation by intra-venous immunoglobin (IVIg). (a-b) Establishing baseline transduction performance of AAV variants in HeLa cells, by flow cytometry. (a) The read-out is percent of eGFP positive alive (DAPI negative) HeLa cells following transduction at the indicated doses. (b) The read-out is mean fluorescent intensity (MFI) of alive (DAPI negative) HeLa cells following transduction at the indicated doses. (c) Transduction of HeLa cells with the indicated AAV variants following incubation the indicated IVIg concentrations. AAV2 and AAV2-7m8 doses were 500 vg/cell, all other variants were used at 5,000 vg/cell to keep the approximate number of transduced cells consistent.


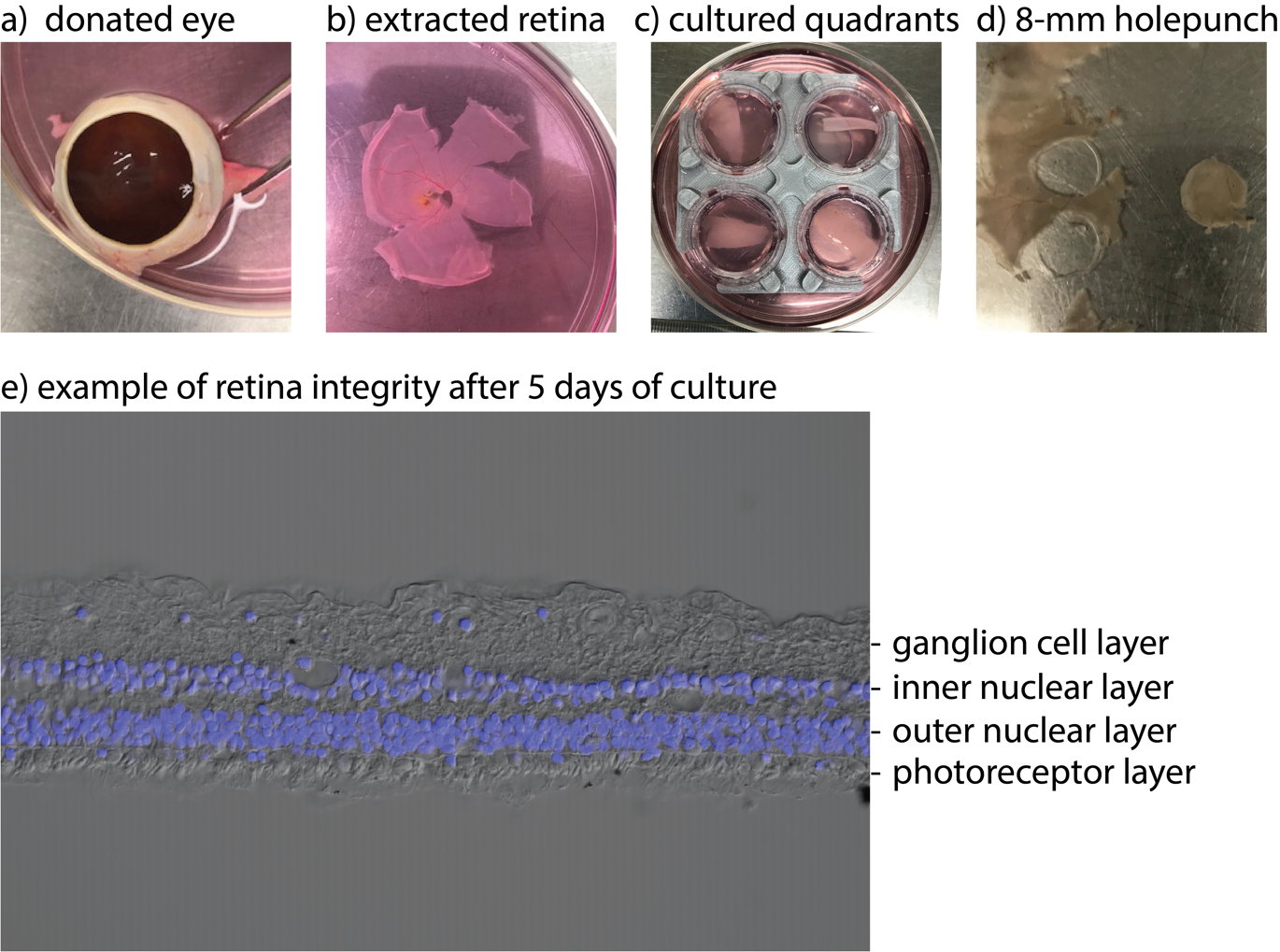


# Supplementary Figure 7. Retina explant preparation and tissue integrity. (a) Eye cup as it arrives from NSW Eye Bank. (b) Extracted and isolated human retina. (c) 8-mm holepunch used for floating explant cultures and interphase explant cultures for immunofluorescence analysis. (d) Interphase culture of retina quadrants for capsid selection and NGS analysis. (e) Representative image of human retina after 5 days of interphase culture.

**References**

1: Atchison, R. W., et al. (1965). "Adenovirus-Associated Defective Virus Particles." Science **149**(3685): 754.

2: Hoggan, M. D., et al. (1966). "Studies of small DNA viruses found in various adenovirus preparations: physical, biological, and immunological characteristics." Proc Natl Acad Sci **55**(6): 1467.

3: Rutledge, E. A., et al. (1998). "Infectious clones and vectors derived from adeno-associated virus (AAV) serotypes other than AAV type 2." Journal of virology **72**(1): 309-319.

4: Parks, W. P., et al. (1967). "Physical Assay and Growth Cycle Studies of a Defective Adeno-Satellite Virus." Journal of Virology **1**(1): 171.

5: Bantel-Schaal, U. and H. Zur Hausen (1984). "Characterization of the DNA of a defective human parvovirus isolated from a genital site." Virology **134**(1): 52-63.

6: Gao, G.-P., et al. (2002). "Novel adeno-associated viruses from rhesus monkeys as vectors for human gene therapy." Proc Natl Acad Sci **99**(18): 11854.

7: Gao, G., et al. (2004). "Clades of Adeno-Associated Viruses Are Widely Disseminated in Human Tissues." Journal of Virology **78**(12): 6381.

8: Mori, S., et al. (2004). "Two novel adeno-associated viruses from cynomolgus monkey: pseudotyping characterization of capsid protein." Virology **330**(2): 375-383.

9: Schmidt, M., et al. (2008). "Adeno-Associated Virus Type 12 (AAV12): a Novel AAV Serotype with Sialic Acid- and Heparan Sulfate Proteoglycan-Independent Transduction Activity." Journal of Virology **82**(3): 1399.

10: Schmidt, M., et al. (2008). "Molecular Characterization of the Heparin-Dependent Transduction Domain on the Capsid of a Novel Adeno-Associated Virus Isolate, AAV(VR-942)." Journal of Virology **82**(17): 8911.

11: Gao, G., et al. (2003). "Adeno-associated viruses undergo substantial evolution in primates during natural infections." Proc Natl Acad Sci **100**(10): 6081.

12: Cabanes-Creus, M., et al. (2020). "Restoring the natural tropism of AAV2 vectors for human liver." Science Translational Medicine **12**(560): eaba3312.

13: Asokan, A., et al. (2010). "Reengineering a receptor footprint of adeno-associated virus enables selective and systemic gene transfer to muscle." Nature Biotechnology **28**(1): 79-82.

14: Zinn, E., et al. (2015). "In Silico Reconstruction of the Viral Evolutionary Lineage Yields a Potent Gene Therapy Vector." Cell Rep **12**(6): 1056-1068.

15: Grimm, D., et al. (2008). "In Vitro and In Vivo Gene Therapy Vector Evolution via Multispecies Interbreeding and Retargeting of Adeno-Associated Viruses." Journal of Virology **82**(12): 5887.

16: Lisowski, L., et al. (2014). "Selection and evaluation of clinically relevant AAV variants in a xenograft liver model." Nature **506**: 382.

17: Paulk, N. K., et al. (2018). "Bioengineered Viral Platform for Intramuscular Passive Vaccine Delivery to Human Skeletal Muscle." Molecular Therapy - Methods & Clinical Development **10**: 144-155.

18: Paulk, N. K., et al. (2018). "Bioengineered AAV Capsids with Combined High Human Liver Transduction In Vivo and Unique Humoral Seroreactivity." Molecular Therapy **26**(1): 289-303.

19: Pekrun, K., et al. (2019). "Using a barcoded AAV capsid library to select for clinically relevant gene therapy vectors." JCI Insight **4**(22).

20: Dalkara, D., et al. (2013). "In Vivo–Directed Evolution of a New Adeno-Associated Virus for Therapeutic Outer Retinal Gene Delivery from the Vitreous." Sci Transl Med **5**(189): 189ra176.

21: Chan, K. Y., et al. (2017). "Engineered AAVs for efficient noninvasive gene delivery to the central and peripheral nervous systems." Nature Neuroscience **20**: 1172.

22: Tervo, D., et al. (2016). "A Designer AAV Variant Permits Efficient Retrograde Access to Projection Neurons." Neuron **92**(2): 372-382.

23: Cabanes-Creus, M. (2019). "Novel AAV engineering technology: identification of improved AAV variants for gene addition and genome engineering in primary human cells." Institute of Child Health, University College London; **PhD** **Thesis** <https://discovery.ucl.ac.uk/id/eprint/10071599>.

24: Cabanes-Creus, M., et al. (2020). "Attenuation of Heparan Sulfate Proteoglycan Binding Enhances In Vivo Transduction of Human Primary Hepatocytes with AAV2." Molecular Therapy - Methods & Clinical Development **17**: 1139-1154.

25: Cabanes-Creus, M., et al. (2022). "Novel human liver-tropic AAV variants define transferable domains that markedly enhance the human tropism of AAV7 and AAV8." Molecular Therapy - Methods & Clinical Development **24**: 88-101.

26: Westhaus, A., et al. (2022). "AAV-p40 bioengineering platform for variant selection based on transgene expression." Human Gene Therapy **33**(11-12): 664-682.
